# Predicting drug combination response surfaces

**DOI:** 10.1038/s44386-024-00004-z

**Published:** 2025-02-03

**Authors:** Riikka Huusari, Tianduanyi Wang, Sandor Szedmak, Tero Aittokallio, Juho Rousu

**Affiliations:** 1 Department of Computer Science, Aalto University, P.O. Box 11000 (Otakaari 1B), FI-00076 Espoo, Finland; 2 Institute for Molecular Medicine Finland (FIMM), HiLIFE, University of Helsinki, Helsinki, FI-00290 Finland; 3 Department of Cancer Genetics, Institute for Cancer Research, Oslo University Hospital, N-0310 Oslo, Norway; 4 Oslo Centre for Biostatistics and Epidemiology (OCBE), Faculty of Medicine, University of Oslo, N-0317 Oslo, Norway

**Keywords:** High-throughput screening, Virtual screening

## Abstract

Prediction of drug combination responses is a research question of growing importance for cancer and other complex diseases. Current machine learning approaches generally consider predicting either drug combination synergy summaries or single combination dose-response values, which fail to appropriately model the continuous nature of the underlying dose-response combination surface and can lead to inconsistencies when a synergy score or a dose-response matrix is reconstructed from separate predictions. We propose a novel prediction method, comboKR, that directly predicts the continuous drug combination response surface for a drug combination. The method is based on a powerful input–output kernel regression technique and functional modelling of the response surface. ComboKR belongs to the family of functional output regression methods, where the prediction target is a function, in our case, a non-linear parametric surface. Our method thus avoids predicting discretized forms of the target as scalars, vectors or matrices, and therefore provides better interpolation and extrapolation along the surfaces. As an important part of our approach, we develop a novel normalisation between response surfaces that standardises the heterogeneous experimental designs used to measure the dose-responses, and thus allows training the method with data measured in different laboratories. Our experiments on two predictive scenarios and using two combination datasets highlight the suitability of the proposed approach especially in the traditionally challenging setting of predicting combination responses for new drugs not available in the training data.

## Introduction

Drug combinations are increasingly used for treatment of various diseases, especially blood cancers and solid tumours^1–3^. In contrast to monotherapies, combination therapies offer the advantages in overcoming intrinsic and acquired resistance in cancer treatment, enhancing drug responses via synthetic lethality, and reducing unwanted side-effects by lowering the dose of individual drugs in the combination^4,5^.

In pre-clinical stages, drug combinations are typically measured in cell lines using dose-response assays. High-throughput screening enables one to measure the responses of pairwise drug combinations at a few selected concentrations of the two drugs (e.g., 5 × 5 or 8 × 8 dose-response matrices)^6^. There are several efforts to conduct large-scale drug combination screens in various cancer types^7–9^, which have resulted either in fully or partially measured dose-response matrices.

The synergistic effects of drug combinations are often evaluated by summary synergy scores, calculated by the divergence between the measured drug combination responses and the expected non-interaction responses of the single drugs over the full matrix^6^. Multiple synergy models have been proposed to score such divergence based on different assumptions of the expected non-interaction response, for example, the highest single agent (HSA) model^10^, Bliss independence model^11^ and Loewe additivity model^12^. However, there are disagreements in terms of synergy when using different synergy models, due to large differences in drug concentrations and maximum response values across studies^13^.

Since drug combination synergy is evaluated based on multi-dose combination responses, often tested in multiple cancer cell lines with distinct oncogene addictions, large-scale screening of combination effects is required for the systematic discovery of new effective and selective combinations. However, to screen pairwise combinations among 100 drugs at 5 different concentrations in 10 cell lines would already require more than one million experimental tests. To speed up such a resource and time-consuming combination discovery process, machine learning models are needed to narrow down the massive combinatorial search space^14,15^.

A large proportion of current research focuses on the prediction of drug combination synergy rather than dose-response values^15–19^. Some research works focus on the prediction of drug combination responses at selected concentrations^20–23^. A major advantage of predicting directly the dose-combination responses is that different synergy models can be applied to the predicted dose-response matrices in post-analysis, making the prediction task independent of a specific synergy metric. One such prediction method is the comboLTR^23^, which predicts directly the scalar-valued dose-combination responses by applying latent tensor-based polynomial regression (LTR)^24^. The drug combination dose-response values can be seen as discrete measurements sampled at different concentration values from a continuous dose-response surface where the response value is a function of drugs’ concentrations. Both synergy score prediction and dose-response prediction can be seen as predictions based on the underlying surfaces: if the full continuous dose-response surface is known, the dose-response matrices can be sampled from the known surface at given concentrations, and the synergy scores can then be derived based on the sampled dose-response matrices. Thus, the direct prediction of a dose–response surface is a more general task than either predicting the single dose–response values or the synergy scores.

Recently, Rønneberg et al.^25^ proposed an approach called PIICM, that considers predicting full response surfaces instead of individual dose-combination responses. They proposed a probabilistic prediction model based on Gaussian process regression where the covariance matrices for pairwise drug interactions are parameterised and learned. Notably, their approach can be interpreted as a matrix completion task on the collected response matrix, as the learning system is based on response data only, without using any additional features (cell line or drug features). This means, however, that the approach can not be expected to adapt to settings where a response surface in a test set would contain a drug not seen in training data. This more challenging new drug scenario is important in practical applications since one cannot assume that responses of all the drugs of interest would been already tested before either individually or in combination.

In this work, we propose a novel approach for predicting directly the full continuous drug combination dose–response surfaces with a kernel-based functional output prediction model, called comboKR. In contrast to the PIICM model^25^, comboKR is based on an inductive learning approach, which predicts the drug combination response surface from input drug features that are easily available from drug databases. Such an inductive machine learning approach is based on the assumption that similar drugs have similar combination surfaces (i.e. if drugs *d*_1_ and ${d}_{1}^{* }$ are similar, so are the combination surfaces of the pairs (*d*_1_, *d*_2_) and $({d}_{1}^{* },{d}_{2})$). Moreover, to overcome the practical issues arising from the heterogeneous experimental design often used in drug combination response measurements, we propose a novel normalisation scheme for comparing drug interaction surfaces. The main goal of the normalisation scheme is to align the dose-response surfaces to be centred around the area where the response changes rapidly as concentrations change. We demonstrate that with comboKR the massive chemical space can be exploited efficiently toward finding novel effective drug combinations beyond the given drug set with known measured responses. To summarise, our contributions are as follows:

- We propose an accurate approach to drug combination response prediction that predicts the full continuous drug combination response surfaces instead of individual dose-response or synergy score values.
- Our surface-valued regression approach takes advantage of a novel normalisation scheme between drug response surfaces that solves issues arising from the heterogeneous experimental designs between and within combination studies.
- Important for novel drug combination discovery, our proposed method can be applied to new drug settings without the need to re-train the model or experimentally measure each drug response beforehand: only functions of the monotherapy responses are required.
- In comparison to the baseline LTR method^26^ and another surface-valued prediction approach, PIICM^25^ (applicable only within simple predictive scenarios), we show that comboKR achieves superior results, especially in the more challenging predictive scenario, where testing is performed on drugs not available in the training stage.

## Overview of ComboKR

We propose a new model, comboKR, to predict the drug combination response surface for a given drug pair in a cell line. In this inductive learning approach, the predictions are made based on the drug features, that can be collected from some database. Our method also uses a paremetrised function to model the drug combination surfaces—in our experiments, we chose to use the BRAID model^27^. With this model, we additionally take advantage of the more abundant monotherapy response data and assume that for the drugs for which a combination surface is being predicted, we know the monotherapy response model.

In this section, we first briefly introduce our predictive framework, after which we discuss the BRAID model, and how to compute similarities between two surface functions.

### Surface-valued regression

Our focus is on learning to predict the full, continuous drug interaction response surfaces $y\in {\mathcal{Y}}$ for the drug pairs $({d}_{1},{d}_{2})\in {\mathcal{X}}$ in a given cell line—a challenging structured output prediction problem. This is an especially difficult prediction task, since practically each output—a surface—in any such data set is sampled in part or fully from different sets of concentrations than the others, and therefore the dose-combination response matrices are not directly comparable. To solve the problem, we consider adapting an approach that has sometimes been referred to as generalised kernel dependency estimation (KDE)^28^ or input–output kernel regression (IOKR)^29,30^.

**Fig. 1:**
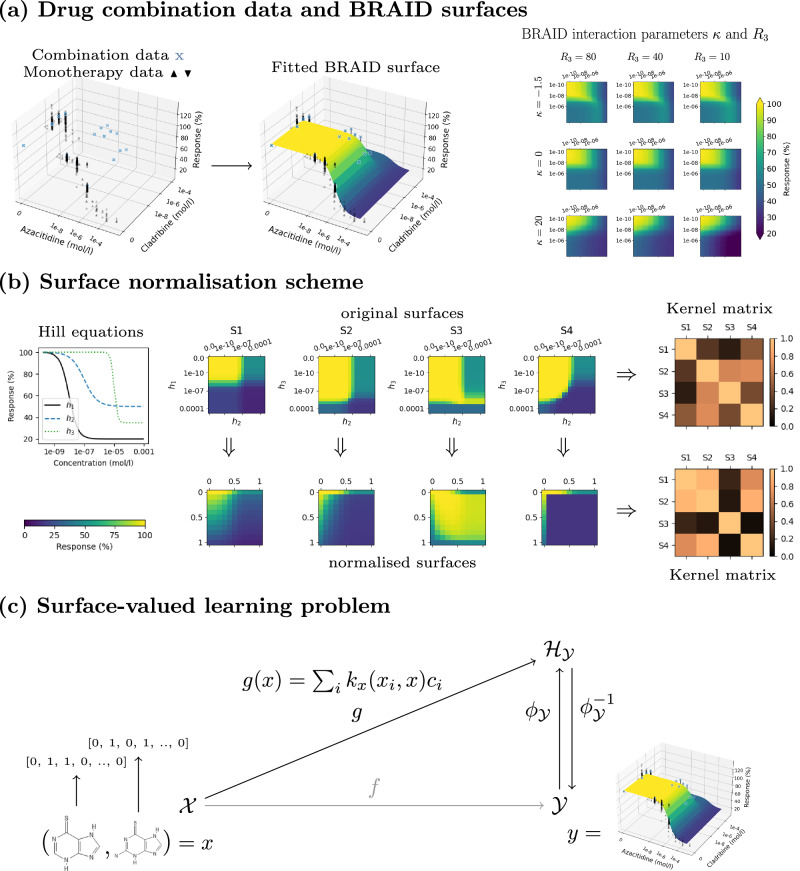
Overview to the proposed approach. **a** The drug combination data and associated monotherapy responses are used to fit a parametric surface on each dose-response matrix. The BRAID surface model uses the Hill equations of the two drugs, as well as two interaction parameters to model different types of response surfaces. **b** Illustration of surface normalisation resulting in different similarities. The surfaces *S*1 and *S*2 are computed with *κ* = 0 (neutral), and *S*2 and *S*4 with *κ* = −1.5 (extreme antagonism) and *κ* = 25 (extreme synergism), respectively. **c** Finally, a surface-valued prediction problem is formulated and solved with the output kernel learning-style approach, where the output data is mapped with the help of a suitable kernel ${k}_{y}:{\mathcal{Y}}\times {\mathcal{Y}}\to {\mathbb{R}}$ to RKHS ${{\mathcal{H}}}_{{\mathcal{Y}}}$ (*ϕ*_*y*_ is the associated feature map: ${k}_{y}({y}_{1},{y}_{2})={\langle {\phi }_{y}({y}_{1}),{\phi }_{y}({y}_{2})\rangle }_{{{\mathcal{H}}}_{{\mathcal{Y}}}}$).

In a nutshell, our proposed model for surface-valued prediction relies on a vector- or function-valued kernel ridge regression (KRR) problem, obtained by mapping the surfaces to the reproducing kernel Hilbert space (RKHS) ${{\mathcal{H}}}_{{\mathcal{Y}}}$ associated with kernel ${k}_{y}:{\mathcal{Y}}\times {\mathcal{Y}}\to {\mathbb{R}}$ (see Fig. 1c). Now, instead of solving directly the structured prediction problem $f:{\mathcal{X}}\to {\mathcal{Y}}$, the learning problem has been cast as vector-valued one to learn $g:{\mathcal{X}}\to {{\mathcal{H}}}_{{\mathcal{Y}}}$, after which the prediction to ${{\mathcal{H}}}_{{\mathcal{Y}}}$ is mapped back to ${\mathcal{Y}}$ (pre-image problem). Using the closed-form solution for the KRR and assuming a normalised output kernel *k*_*y*_, the final optimisation problem to obtain predictions can be written as

1 $$f(x)=\mathop{{\mathrm{arg}}\,{\mathrm{max}}}\limits_{y\in {\mathcal{Y}}}{k}_{y}(y,Y){({{\bf{K}}}_{x}+\lambda {{\bf{I}}}_{n})}^{-1}{k}_{x}(X,x).$$

Here **I**_*n*_ is *n* × *n* identity matrix, and *λ* is the regularisation parameter of the kernel ridge regression model. **K**_*x*_ stands for the *n* × *n* kernel matrix collecting all values of kernel evaluations between pairs of drugs *k*_*x*_(*x*_*i*_, *x*_*j*_), *i*, *j* = 1, …, *n* and each *x*_*i*_ stands for a (ordered) pair of drugs, ${x}_{i}={({d}_{1},{d}_{2})}_{i}$. In this work, we have chosen to use as *k*_*x*_ the Tanimoto similarity computed from the MACCS fingerprints; however our framework is general and adaptable to other input data representations with suitable kernel. The shorthand *k*_*y*_(*y*, *Y*) refers to the vector [*k*_*y*_(*y*, *y*_1_), …, *k*_*y*_(*y*, *y*_*n*_)] with ${\{{y}_{i}\}}_{i = 1}^{n}=Y$ the outputs of the training set; *k*_*x*_(*X*, *x*) is defined analogously.

Problem (1) is ill-posed in general. When this is the case, it is most often solved by restricting the search for maximum value over a candidate set *C*_*y*_ instead of the full output space ${\mathcal{Y}}$. Every element in the set is tried out, and the one giving the maximum value for (1) is chosen as the final prediction.

### BRAID surfaces

Our learning approach considers full dose-response surfaces as the outputs of the learning problem. Yet, the dose–response data is collected via factorial experimental design by measuring response values from varying dose concentrations, resulting in data sets where these surfaces are represented with matrices collecting the measurements. Starting from the famous Loewe additivity^12^ and Bliss independence^11^, various approaches to modelling drug interactions have been proposed^27,31–35^. In this work, in order to obtain the continuous form of the surface, we use the bivariate response to additive interacting doses (BRAID) drug interaction model^27^ that builds on the Hill equation^36,37^ and is motivated by the Loewe additivity principle. We fit this function to each of the drug pair combinations. The model is intuitive, as it uses the Hill equation parameters of the two drugs, in addition to two interaction parameters. More concretely, the function depends on the following parameters:

- Four response parameters: the baseline response in the absence of drugs, *R*_0_; the maximal responses of drugs 1 and 2 as *R*_1_ and *R*_2_; and optionally also the maximal combination response *R*_3_. The *R*_3_ parameter is not present in the original BRAID model introduced in ref. ^27^, but it is present in the implementation^38^.
- Hill equation slope parameters for both drugs: *τ*_1_ and *τ*_2_.
- Half maximal effective concentration (EC_50_) for the two drugs: EC_1_ and EC_2_.
- The interaction parameter $\kappa \in \left.\right]-2,\infty \left.\right]$: *κ* < 0, *κ* = 0, *κ* > 0 for antagonism, additivity or synergy, respectively (as illustrated in Fig. 1a).

Now, the BRAID function for drugs 1 and 2 applied in dose concentrations *c*_1_ and *c*_2_ is written as

$${{BRAID}}({c}_{1},{c}_{2})={R}_{0}+\frac{{R}_{1}-{R}_{0}}{1+{\tilde{D}}_{12}^{-\sqrt{{\tau }_{1}{\tau }_{2}}}}$$

where

$${\tilde{D}}_{12}=\,{\tilde{D}}_{1}^{1/\sqrt{{\tau }_{1}{\tau }_{2}}}+{\tilde{D}}_{2}^{1/\sqrt{{\tau }_{1}{\tau }_{2}}}+\kappa \sqrt{{\tilde{D}}_{1}^{1/\sqrt{{\tau }_{1}{\tau }_{2}}}{\tilde{D}}_{2}^{1/\sqrt{{\tau }_{1}{\tau }_{2}}}}$$

$${\tilde{D}}_{1}=\,{\left(\frac{{c}_{1}}{E{C}_{1}}\right)}^{{\tau }_{1}},\,{\tilde{D}}_{2}=\,\frac{\left(\frac{{R}_{2}-{R}_{0}}{{R}_{1}-{R}_{0}}\right){\left(\frac{{c}_{2}}{E{C}_{2}}\right)}^{{\tau }_{2}}}{1+\left(1-\frac{{R}_{2}-{R}_{0}}{{R}_{1}-{R}_{0}}\right){\left(\frac{{c}_{2}}{E{C}_{2}}\right)}^{{\tau }_{2}}}.$$

We note that in this formula it is assumed that one of the drugs is the weaker one, and the other stronger. In the practical implementation^39^ either of the drugs could be the stronger one, resulting to a slightly more complicated formula. The implementation provides methods to fit the BRAID functions to data.

Due to the scarcity of the combination data (for example only 3 × 3 measurements for each combination in the NCI-ALMANAC dataset), to aid with the fitting process we additionally include the more abundant monotherapy data available from other sources if possible. We illustrate this in Fig. 1a.

The final optimisation problem of our model (Eq. (1)) is solved with the candidate set optimisation. Here, domain knowledge can be exploited in making the most suitable candidates: we make the natural assumption that the monotherapy equations for the two drugs are known. Compared to drug combination response data, monotherapy data is easier to collect, and many databases already exist for those measurements. With the monotherapy equations known, generating the candidate sets with the BRAID surface model boils down to generating suitable parameter combinations for the interaction parameters *R*_3_ and *κ*.

### Kernels between surfaces

Our surface-valued prediction approach relies on having a kernel, *k*_*y*_, defined between the drug interaction surfaces. We choose to use the Gaussian (or RBF) kernel, defined as ${k}_{{\rm {RBF}}}(z,{z}^{{\prime} })={{\rm {e}}}^{-\gamma \parallel z-{z}^{{\prime} }{\parallel }^{2}}$ for some vectors *z* and ${z}^{{\prime} }$. At first glance, it might seem attractive to use the (vectorised) dose-response matrices directly available in drug combination datasets in this kernel. However, even if those matrices were of the same size, most often the drug doses used to measure drug combination responses are widely different between any two surfaces. Thus, these measurements cannot be directly compared to each other. We instead assume that we have functions ${S}_{i}:{\mathbb{R}}\times {\mathbb{R}}\to {\mathbb{R}}$, mapping two dose concentrations to a response value, parameterising the drug interaction surfaces available—in our work we obtain these from the BRAID interaction model. We can now fix a set of concentrations for all drugs, $C=[[{c}_{1},{c}_{1}^{{\prime} }],[{c}_{1},{c}_{2}^{{\prime} }],\ldots ,[{c}_{N},{c}_{N}^{{\prime} }]]$, and compare any two surfaces in for example RBF kernel with

$${k}_{y}({S}_{A},{S}_{B})=\exp (-\gamma \parallel {S}_{A}(C)-{S}_{B}(C){\parallel }_{{\rm {F}}}^{2}),$$

where ${S}_{A}:{\mathbb{R}}\times {\mathbb{R}}\to {\mathbb{R}}$ and ${S}_{B}:{\mathbb{R}}\times {\mathbb{R}}\to {\mathbb{R}}$ are response surface functions (e.g. BRAID). Both of them are defined for a pair of drugs on a cell line and are here queried with some concentration values, returning the response values at those dose combinations. Our shorthand notation (*S*_1_(*C*) and *S*_1_(*C*)) evaluates these functions at all concentration pairs in the grid defined by *C*, which, after properly reshaping, gives two *N* × *N* matrices in the Frobenius norm evaluation in the kernel.

However, this straightforward approach still has limitations. Different drugs often have different effective concentrations, meaning that two combination surfaces expressing very similar interaction profiles (e.g. similar levels of synergy) might be shifted in relation to each other so that the kernel evaluation results in non-intuitive values. To overcome this issue, we propose normalisation over the dose-concentration values to an effective concentration range [0, 1] using the Hill equation, where 0 corresponds to concentrations that have no effect on the cell growth, 0.5 indicates EC_50_ concentration, and 1 corresponds to concentrations with a maximal response. We denote kernel acting on normalised surfaces as ${\tilde{k}}_{y}$.

With this normalisation, problems related to shifted surfaces are decreased, and the kernel ${\tilde{k}}_{y}$ shows more realistically the differences in interaction pattern (i.e. if the combination is synergistic or antagonistic). We illustrate this difference between the two choices of kernels in Fig. 1b, where surfaces of three types of interactions (*S*2, *S*3 and *S*4) are compared to baseline surface *S*1. The three surfaces differ from *S*1 by one of the Hill functions (*h*_3_ instead of *h*_1_) and the interaction parameter *κ*. Intuitively, the surfaces *S*1 and *S*2 are closest together in the sense that their drug combination interaction effect is very similar (*κ* = 0). However, the Hill equations and especially their EC_50_ values for the first drug are different (equation *h*_1_ vs. *h*_3_), which has resulted in the two surfaces being “shifted” w.r.t. each other. The Frobenius norms between the original surfaces are, in all three cases, large, and *S*1 is judged most similar to *S*3. The normalised comparison can take the shifting into account, and *S*2 is therefore judged to be most similar to *S*1.

## Results

In this section, we computationally validate our proposed surface-valued kernel regression model for drug combination response prediction, comboKR. We consider two drug combination datasets: the NCI-ALMANAC^7^, and O’Neil^40^. We compare our comboKR to a scalar-valued dose-response prediction baseline, LTR^26^, and to another surface-valued prediction model, PIICM^25^. For datasets where PIICM does not scale well, we compare to a model derived from it—we call this PIICM^*^ (see the “Methods” section for details). Due to the computational cost of comboKR arising from the use of kernel matrices, we consider all the cell lines as independent data sets, and train and test on them separately—a challenging setting that often arises in real-world personalised medicine applications. For comboKR we consider both original and normalised output kernels and denote as “comboKR raw” or “comboKR r.” the version with the original, simpler kernel, while “comboKR” denotes version with our normalisation scheme.

The results presented here are obtained from two representative predictive scenarios: new drug and new combo. New combo refers to the case when the test set consists of new drug combinations—however, all the drugs are available in the training set as parts of other combinations. New drug is more realistic, but also more challenging scenario, where the drug combinations in the test set always contain a drug that has not been present in any of the combinations in the training set.

### Overview of the results

**Fig. 2:**
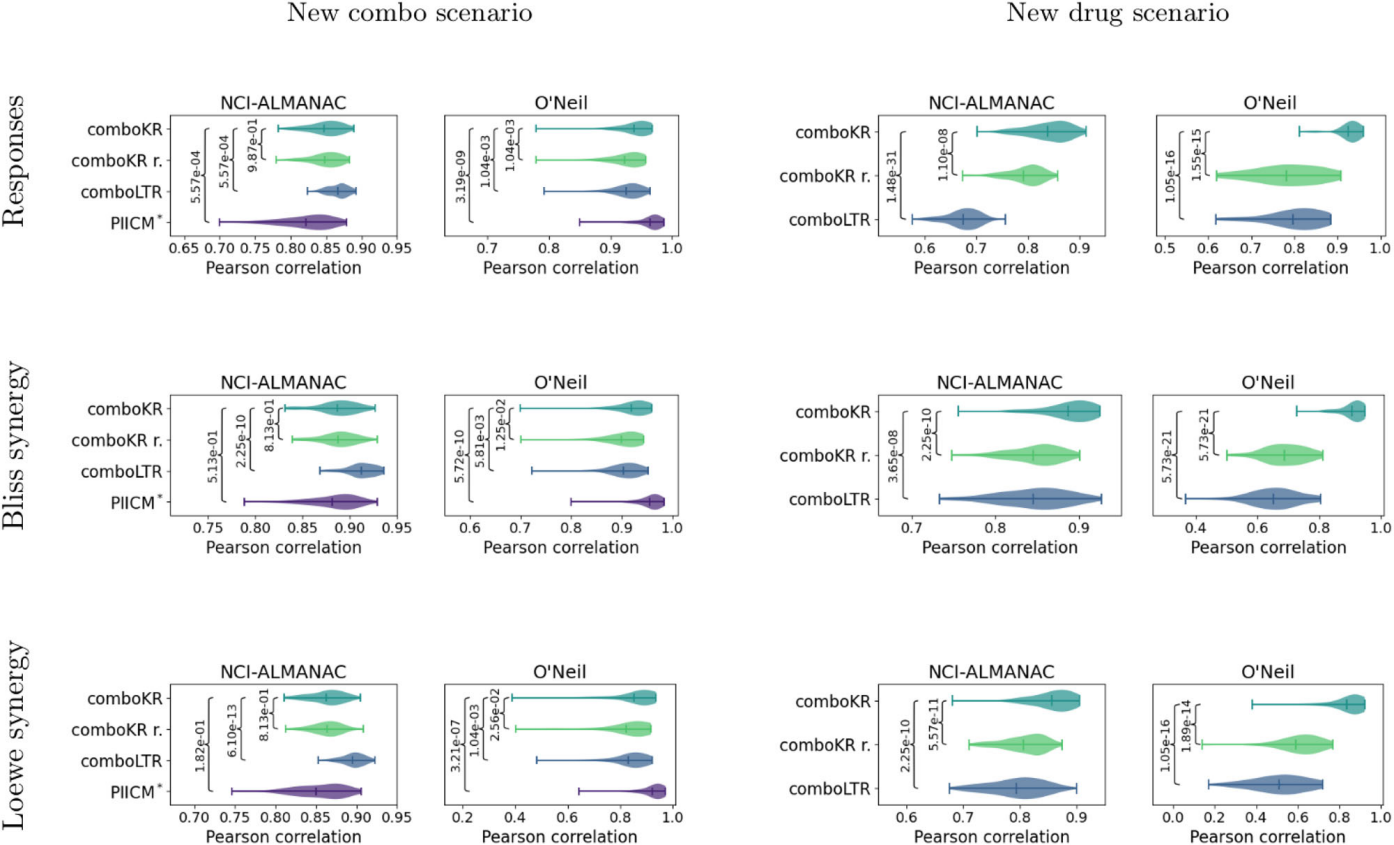
Violin plots of Pearson correlations over the cell lines in the two prediction settings (NCI-ALMANAC: 60 cell lines; O’Neil: 39 cell lines). The vertical lines in the plots highlight the mean and the extrema. First row of plots shows a correlation between original and predicted combination responses. Additionally, synergy scores (Bliss and Loewe) were calculated from both ground truth measurements as well as for the predictions. The two other rows show correlations of these synergy scores. Statistical *p*-values from two-sided Kolmogorov–Smirnov test is shown for all the competing methods compared to ComboKR (full pairwise results are available in Supplementary material).

**Fig. 3:**
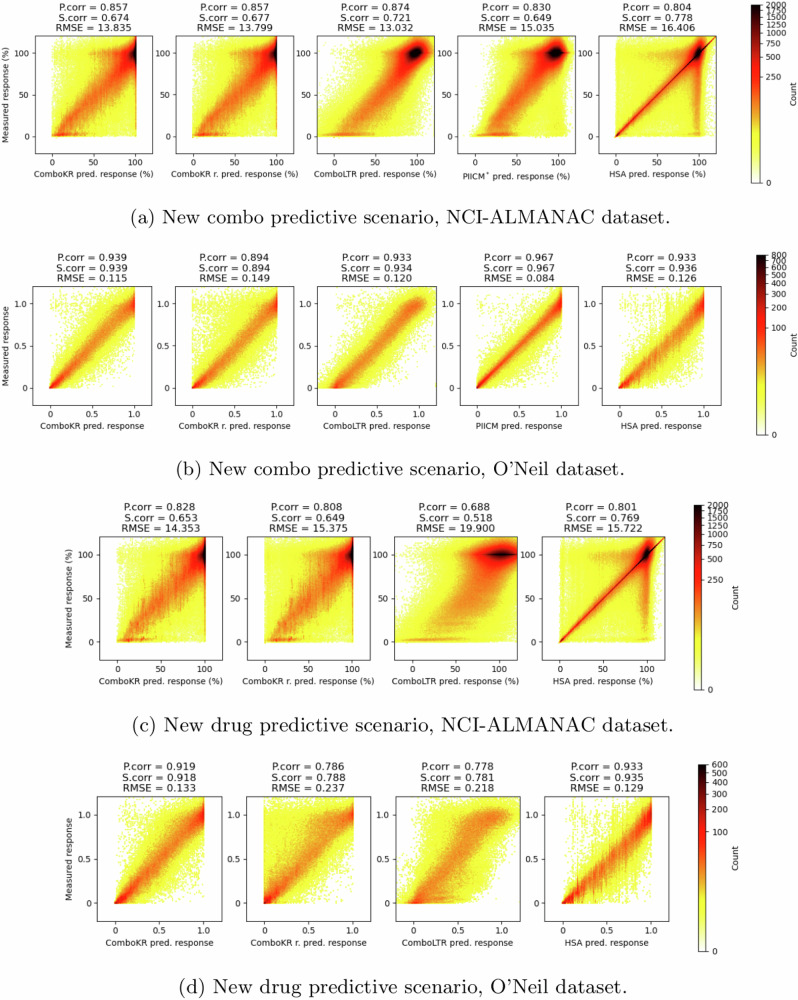
Density plots of predictions and ground truth measurements over all cell lines for the two predictive scenarios. The titles of the plots indicate the Pearson correlation, Spearman correlation and root mean squared error (RMSE) between the predicted and the measured response values. The panels **a** and **c** show results for NCI-ALMANAC dataset, while **b** and **d** show them for O’Neil. Panels **a** and **b** show results of the new combo scenario, and **c** and **d** show results in the more challenging new drug scenario.

We present the overall Pearson correlations averaged over the cell lines between the predicted values and ground truth measurements, as well as between the Bliss and Loewe synergies calculated from them (Fig. 2). For the response values, we also display density plots in Fig. 3 where include additionally HSA baseline^10^. In the easier new combo scenario on the NCI-ALMANAC dataset, the scalar-valued prediction approach, LTR, slightly outperforms all the surface-valued ones. With O’Neil data the PIICM method slightly outperforms others. Yet, the differences between the methods are relatively small. However, in the more challenging new drug scenario, the situation reverses, and the surface-valued approaches mostly outperform LTR. In this more challenging setting, comboKR has a clear advantage over the other models, especially when using the concentration normalised surface kernels. PIICM inherently requires all the drugs (and cell lines) in the test set and also in training, and thus cannot be applied in this setting. With the NCI-ALMANAC dataset, all the predictive models outperform the HSA baseline in the easier scenario, while comboKR continues to outperform it also in the more difficult one. With the smaller O’Neil dataset, all the results are closer to the baseline.

It can be seen from Fig. 3 that the surface-valued models can offer improvements over the scalar-valued model in predicting the extreme response values that are more rare in the data. The LTR model focuses on the predictions of the more abundant, higher response values, and easily overshoots the predictions of the lower response values. While this is easier to see in the new drug predictive scenario between comboKR and LTR, the same behaviour is already present in the new combo setting. With NCI-ALMANAC data, the other surface-valued method, PIICM^*^, predicts the extreme response values better than LTR but not as well as the comboKR approaches. However, with O’Neil data—with which PIICM was originally evaluated with^25^—PIICM outperforms the other methods.

In supplementary material, we show additional results for the correlations with the fitted BRAID surfaces, as well as full pairwise results on the statistical significance of the differences between results displayed in Fig. 2.

### Performance over tissue and drug combination types

Both NCI-ALMANAC’s and O’Neil dataset’s cell lines originate from multiple tissue types: nine types in NCI-ALMANAC, six in O’Neil. Similarly, the tested drugs belong to three drug groups (chemotherapy, targeted and other). Details of these groupings can be found from the supplementary material.

**Fig. 4:**
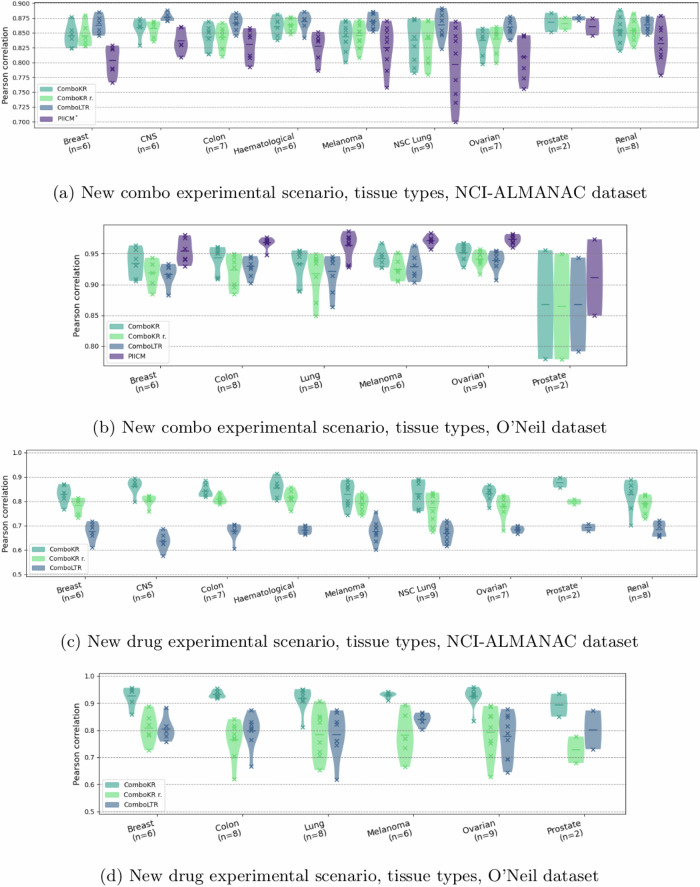
Distributions of Pearson correlations of the drug-dose response prediction on the different tissues (see supplementary material for details) in the two predictive scenarios, for the comboKR variants and LTR, as well as for PIICM variants in the new combo scenario. The vertical lines in the plots highlight the mean. The violins with fewer than ten elements indicate the number of elements in parenthesis of the horizontal axis label and additionally include also the individual results, marked with crosses. The panels **a** and **c** show results for NCI-ALMANAC dataset, while **b** and **d** show them for O’Neil. Panels **a** and **b** display results of the new combo scenario, and **c** and **d** display the results in the more challenging new drug scenario.

**Fig. 5:**
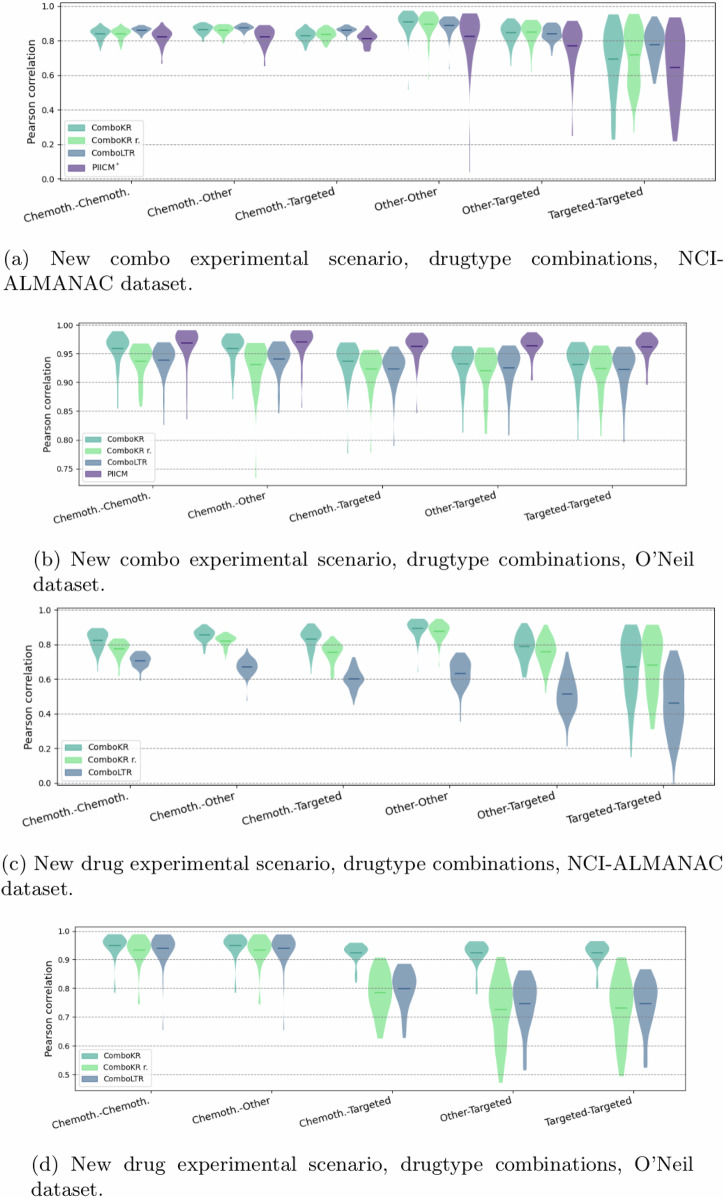
Distributions of Pearson correlations of the drug-dose response prediction on the different drug type combinations (see supplementary material for details) over the 60 (NCI-ALMANAC) or 39 (O’Neil) cell lines in the two predictive scenarios. The vertical lines in the plots highlight the mean. For O’Neil data, there is no “Other-Other” drug-type combination. The panels **a** and **c** show results for NCI-ALMANAC dataset, while **b** and **d** show them for O’Neil. Panels **a** and **b** display results of the new combo scenario, and **c** and **d** display the results in the more challenging new drug scenario.

We investigated and compared in more detail the performance of the methods on these different tissue types and different drug type combinations in the two predictive settings (Figs. 4 and 5). As before, the LTR method performs slightly better than the comboKR approaches in the easier predictive scenario with the NCI-ALMANAC dataset, while PIICM outperforms others in this setting with the O’Neil dataset. Yet, looking at drug-type combination results, it can be seen that the PIICM has less advantage with chemotherapy drugs, with which comboKR performs similarly to PIICM. In the new drug scenario, comboKR clearly outperforms comboKR r. and LTR, while with the NCI-ALMANAC dataset also comboKR r. outperforms LTR. While the difference between the two comboKR variants is larger in the challenging new drug setting, also in the easier new combo setting on the O’Neil dataset, comboKR already outperforms the simpler comboKR r. approach in some cell lines and drug type combinations.

### Performance of predicting individual surfaces

The results so far have focused on the overall performance of the models. Here, we show in Table 1 the results of the pairwise comparison of individual surface predictions. The table reports the average percentages (averaged over the cell lines) of how often a method achieved a better prediction for the surfaces in the test set, w.r.t. mean squared error, compared to the other methods (the comparison is always pairwise).

**Table 1:** Pairwise comparison of the methods

The table reports average percentages over all cell lines. How often did the method on row give a better prediction for a surface, w.r.t. mean squared error, than the method on column; rows with high values (darker colouring; similarly columns with low values with lighter colouring) indicate better performance for the method. The two comboKR versions with original and normalised surface kernels use the same candidate sets, and so their predictions might be identical, resulting in ties in the rankings. Tie counts are not included in the tables, so cells marked with asterisk do not add to 100%.

Similarly to the previous results, also here it can be observed that the differences between the methods are small in the easier new combo predictive scenario (Table 1a and c). The new drug predictive scenario (Table 1b and d) highlights again the benefits of the surface-valued methods: LTR obtains most often the worst prediction for a surface. The results again highlight how using the normalised surface kernels outperforms the basic comboKR r. approach. Notably the amount of identical predictions between the two comboKR versions is almost half the amount of that in the new combo setting.

An advantage to our method is that comboKR predicts a full continuous interaction surface, from which one can sample any dose-concentration pairs to obtain dose-specific predictions or summary synergy scores. Contrary to this, the baseline LTR accurately predicts only those concentrations measured in the drug response assay separately, which can lead to predictions that do not follow a smooth interaction pattern. We illustrate this in Supplementary material.

## Discussion

In this work, we have investigated the drug combination response prediction problem from the point of view of predicting entire drug combination surfaces, instead of predicting individual response values. We propose an approach based on kernel methods, which when combined with a novel surface normalisation scheme, overcomes issues arising from the heterogeneous experimental design used to measure the data. We show that casting the drug combination response prediction as a structured prediction learning problem can improve predictive performance, especially in traditionally challenging experimental settings. Namely, our method shows great promise especially in the new drug scenario, providing the opportunity to find promising drug combinations that go beyond the limited set of drugs in the training set.

To explore the suitability of our proposed surface-valued learning approach, we performed computational experiments on the NCI-ALMANAC and O’Neil datasets. Of the two predictive scenarios investigated in our experiments, the proposed surface-based approach achieves better predictive performance, especially in the more challenging new drug scenario. Even the more straightforward surface-valued prediction approach outperformed the baseline LTR method on one dataset, but especially the novel concentration normalisation provides significant improvements in this challenging, yet practical setting. In personalised medicine studies, either focusing on individual cell lines or patient samples, one cannot assume that each drug has already been tested in combination with other drugs in often limited training datasets. In addition to outperforming other approaches, the suitability of our proposed comboKR in the new drug scenario is also computationally practical: unlike with the traditional methods, the proposed model does not need to be re-trained to obtain predictions when a new set of drugs is introduced to the test set; predicting only requires the monotherapy response function for the (new) drugs. Moreover, the comboKR predicts a full continuous drug interaction surface, instead of individual values that might not conform to a smooth interaction pattern. Again, this gives a practical advantage: it is easier, based on the predicted surface to experimentally validate the synergy between two drugs by using the surface to determine relevant drug concentrations. Surface sampling can also be used to suggest doses for experimental testing with highest likelihood for revealing synergy between two drugs.

Additionally, we observed that the surface-valued methods, in general, were better suited for predicting extreme response values (Fig. 3). This was most clearly observed in the new drugs setting. The extreme responses are often most informative for identifying synergistic (or antagonistic) interactions between two drugs, so their prediction is critically important for drug combination discovery. Our comparison with the HSA baseline showcases the overall difficulty of drug combination response prediction: even such a simple baseline can perform relatively well, most likely due to the presence of extreme combination responses that can be captured by most models. Indeed, HSA is equal to the concept of independent drug action (IDA) that can explain many of the clinically beneficial drug combinations, even without pre-clinical synergy^3^. Such a simple baseline can thus provide a useful first guess for modelling novel drug interaction patterns.

Our experiments give promising results for using a structured prediction approach in the drug combination response prediction, motivating future research. It will be important to investigate ways to make the ComboKR model more scalable on big high-throughput screening data consisting of millions of data points. A more scalable model would allow more variety in the predictive scenarios: if trained over multiple cell lines, a fully new cell line could be included in the predictive stage. The obvious bottleneck for applications to multiple cell lines is the sampling of the large kernel matrix on inputs, consisting of two Kronecker products: *K*_*c*_⊗*K*_*d*_⊗*K*_*d*_. In addition to efficient algorithms and parallelisation strategies, for example, kernel approximations could be investigated to speed up the computations. Another avenue to pursue would be to follow^41^, and investigate the scalability using or generalising the proposed Kronecker product vec-trick. Finally, it would be interesting to investigate and compare different drug combination surface models. In current work, we have used the BRAID surfaces, but our method could be applied to any model.

## Methods

### Drug combination datasets

In this study, we consider two drug combination datasets: the NCI-ALMANAC^7^, and O’Neil^40^—see Table 2 for a summary of them.

**Table 2:** The amount of cell lines and drugs in the datasets considered in this work

Dataset	#Cell lines	#Drugs	#Surfaces	Measurements
NCI-ALMANAC	60	104	311 527	3 × 3; one set
O’Neil et al	39	38	22 527	4 × 4; four sets

The table reports also the amount and sizes of the drug combination matrices measured in the data.

The O’Neil dataset provides a screen of 38 unique drugs in pairwise combinations on 39 diverse cancer cell lines (see Supplementary material). The combination data consists of 4 × 4 drug combination measurements on the cell lines; in total there are 22,527 of such drug–drug-cell combinations. (We note that the full data size is 22,727^40^, but 22,527 is the amount of the surfaces with measurements on 4 × 4 grid. The other 200 are measured at non-conforming grids.) In this work, the median response values over the four measurement replicats are considered to be the groundtruth responses for the experiments. In addition to the combination data, the dataset contains monotherapy measurements for the drugs on the cell lines, typically on six measurement replicats.

The NCI-ALMANAC dataset provides systematic screening of drug combinations among 104 FDA-approved anticancer drugs on the 60 NCI-60 human tumour cell lines covering 9 different tissue types (see Supplementary material). In this dataset, drugs have been screened at either 5 or 3 concentrations, resulting in 5 × 3 or 3 × 3 drug combination dose-response matrices. As the number of larger dose-response matrices is much fewer than 3 × 3 matrices, in order to have consistent dose–response matrix size in method evaluation, the large dose–response matrices were subsampled to 3 × 3 by keeping the entries corresponding to the largest dose concentrations. Monotherapy responses at combination dose concentrations were also included in the NCI-ALMANAC dataset for the 104 drugs, where single-drug responses were measured at various concentrations. Different concentration values were determined for different drugs^7^. Thus, each drug combination response surface is represented by the measured responses by a 4 × 4 matrix containing both combo- and mono-responses. When duplicate measurements of the same drug combination on the same cell line appeared, the median of measurements was taken.

**Fig. 6:**
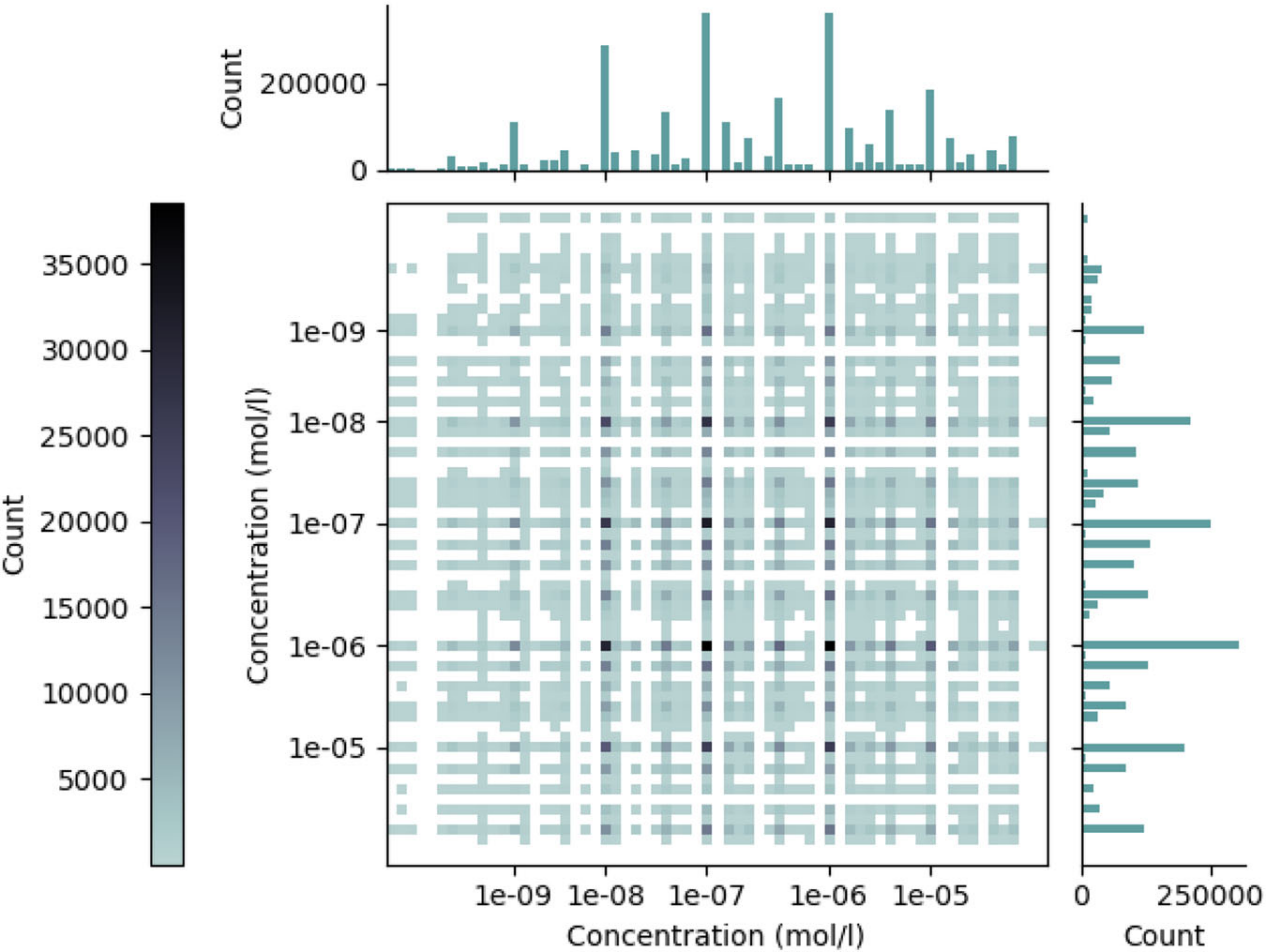
The drug dose combination counts in NCI-ALMANAC dataset. The two axes refer to the two drugs in combination, for which the corresponding dose concentration counts are displayed in the bar plots. The counts of the drug dose combinations are displayed in the matrix, where a darker colour indicates a higher count.

NCI-ALMANAC data has been collected with a standard factorial experimental design. The 3-by-3 (or 4 × 4), dose–response matrices described above are independent of each other in a sense, that the dose levels between experiments (matrices) used to collect the data are not the same. Notably, even if two response matrices both consider the same drug as one factor, the concentrations used in measurements might differ between them. To illustrate this, Fig. 6 shows that here is a large amount of different concentration combinations in the NCI-ALMANAC data. On average, any given dose combination is found from only 0.44% of the drug response matrices. The most common concentration combination is present at 12.4% of the matrices (the full distribution is shown in Supplementary Material). It is very rare that for any two surfaces, all nine concentration combinations would match. Thus, directly comparing any two dose-response matrices in the dataset can very rarely be done.

NCI-ALMANAC reported two endpoints calculated differently by using time zero measurement as reference or not^42^. For the percent growth of cells with time zero as reference (“PercentGrowth” as reported in the dataset), the responses range from −100 to 100. However, for this endpoint, the calculation processes are different when the percent growth of test cells is lower or higher than the time zero measurement of cell growth. Whereas for the percent growth of cells using only control values (“PercentGrowthNoTZ” as reported in the dataset) as a reference, the response calculation is consistent and ranges from 0 to 100. Thus, for simplicity and consistency of data, the endpoint denoted as “PercentGrowthNoTZ” was used as the response value. If there were missing values for the endpoint, it was calculated from the raw measurements reported in the dataset. For O’Neil dataset, we used directly the viability measurements as the responses that were provided in the data^40^. The values mostly range in [0, 1], sometimes higher values than 1 are present.

Our proposed comboKR, as well as the compared PIICM method, rely on accurate surface modelling in the training stage. Since the NCI-ALMANAC drug combination response data only contains three monotherapy measurements for both drugs, the NCI-60 single drug monotherapy response data where typically a drug is measured at multiple concentrations was also integrated as part of the surface model fit procedure, in order to help improve the model performance with a better estimate of monotherapy dose-response functions. We remark that the O’Neil dataset directly includes additional monotherapy viability measurements in addition to the combination data.

To train the machine learning models to predict the dose–response values, we consider the commonly used 166-bit 2D structure MACCS molecular fingerprints as the input data.

### The ComboKR model

Our proposed ComboKR model is an adaptation of an approach that has sometimes been referred to as generalised kernel dependency estimation (KDE)^28^ or input–output kernel regression (IOKR)^29,30^.

In this approach, operator-valued kernels (OvKs) are used to solve a regression problem to a reproducing kernel Hilbert space (RKHS) ${{\mathcal{H}}}_{{\mathcal{Y}}}$ induced by a traditional scalar-valued kernel *k*_*y*_ defined for the output data in ${\mathcal{Y}}$. OvKs are associated with vector-valued reproducing kernel Hilbert spaces (vv-RKHSs), containing functions that map the data to the vector-valued (or function-valued) output space. Thus, they are a natural choice to solve for the function $g:{\mathcal{X}}\to {{\mathcal{H}}}_{{\mathcal{Y}}}$.

In this context, the operator-valued kernel is a function ${\mathcal{K}}:{\mathcal{X}}\times {\mathcal{X}}\to {\mathcal{L}}({{\mathcal{H}}}_{{\mathcal{Y}}})$ in which ${\mathcal{L}}({{\mathcal{H}}}_{{\mathcal{Y}}})$ denotes the set of linear operators from ${{\mathcal{H}}}_{{\mathcal{Y}}}$ to ${{\mathcal{H}}}_{{\mathcal{Y}}}$—if the output space ${{\mathcal{H}}}_{{\mathcal{Y}}}$ is finite-dimensional, i.e. ${{\mathcal{H}}}_{{\mathcal{Y}}}={{\mathbb{R}}}^{p}$, then ${\mathcal{L}}({{\mathcal{H}}}_{{\mathcal{Y}}})={{\mathbb{R}}}^{p\times p}$. In practise, the most common operator-valued kernel to use is the separable (or decomposable) kernel, which can be written as ${\mathcal{K}}(x,z)={k}_{x}(x,z){\bf{T}}$ with ${k}_{x}:{\mathcal{X}}\times {\mathcal{X}}\to {\mathbb{R}}$ being a traditional scalar-valued kernel on the input data, and ${\bf{T}}\in {\mathcal{L}}({{\mathcal{H}}}_{{\mathcal{Y}}})$, which in turn is often chosen to be the identity.

Operator-valued kernels generalise the usual scalar-valued kernels, notably also the representer theorem. Thus, the solution to the regularised learning problem considered in the IOKR,

$$g(x)=\mathop{\min }\limits_{f\in {{\mathcal{H}}}_{X}}\mathop{\sum }\limits_{i=1}^{n}{\left\Vert {y}_{i}-g({x}_{i})\right\Vert }^{2}+\lambda \parallel g{\parallel }_{{{\mathcal{H}}}_{X}}^{2},$$

where ${{\mathcal{H}}}_{X}$ denotes the vv-RKHS associated with ${\mathcal{K}}$, can be written as

$$g(x)=\mathop{\sum }\limits_{i=1}^{n}K(x,{x}_{i}){{\bf{c}}}_{i}.$$

Here ${{\bf{c}}}_{i}\in {{\mathcal{H}}}_{{\mathcal{Y}}}$ are the multipliers to be learned. Like in the usual scalar-valued case, the closed-form solution

$${\rm{vec}}({\bf{C}})={({\bf{G}}+\lambda {\bf{I}})}^{-1}{\rm{vec}}(\Phi (Y))$$

can be obtained, in which *Φ*(*Y*) = [*ϕ*_*y*_(*y*_1_), *ϕ*_*y*_(*y*_2_), …, *ϕ*_*y*_(*y*_*n*_)] and **C** = [**c**_1_, **c**_2_, …, **c**_*n*_], both of size *d* × *n* if the size of feature space associated with kernel *k*_*y*_ is denoted with *d*. **G** is the *nd* × *nd* operator-valued kernel matrix with block-wise structure. Now,

$$g(x)={\mathcal{K}}(x,X){\rm{vec}}({\bf{C}})={\mathcal{K}}(x,X){({\bf{G}}+\lambda {\bf{I}})}^{-1}{\rm{vec}}(\Phi (Y)),$$

where $${\mathcal{K}}(x,X)=[{\mathcal{K}}(x,{x}_{1}),\ldots ,{\mathcal{K}}(x,{x}_{n})]$$

After solving for *g*, the final predictions can be obtained from the pre-image problem

$$\mathop{{\mathrm{arg}}\,{\mathrm{min}}}\limits_{y\in {\mathcal{Y}}}\,\parallel {\phi }_{y}(y)-g(x){\parallel }^{2}$$

or from

$$\mathop{{\mathrm{arg}}\,{\mathrm{max}}}\limits_{y\in {\mathcal{Y}}}\,\langle {\phi }_{y}(y),g(x)\rangle$$

if one assumes that the output kernel *k*_*y*_ is normalised, i.e. ${k}_{y}(y,y)=1\,\forall y\in {\mathcal{Y}}$. It is now possible to take advantage of the form of the separable kernel matrix, and the Kronecker product vec-trick, to obtain the final optimisation problem

2 $$f(x)=\mathop{{\mathrm{arg}}\,{\mathrm{max}}}\limits_{y\in {\mathcal{Y}}}{k}_{y}(y,Y){({{\bf{K}}}_{x}+\lambda {{\bf{I}}}_{n})}^{-1}{k}_{x}(X,x).$$

Here **I**_*n*_ is *n* × *n* identity matrix, and **K**_*x*_ stands for the *n* × *n* kernel matrix ${[{k}_{x}({x}_{i},{x}_{j})]}_{i,j = 1}^{n}$. The shorthand *k*_*y*_(*y*, *Y*) with $Y={\{{y}_{i}\}}_{i = 1}^{n}$ the training set outputs, refers to the vector [*k*_*y*_(*y*, *y*_1_), …, *k*_*y*_(*y*, *y*_*n*_)]; *k*_*x*_(*X*, *x*) is defined analogously.

#### Algorithm 1

**The comboKR approach**

**Require** ${{\bf{K}}}_{x}^{{\rm {train}}}\in {{\mathbb{R}}}^{{n}_{{\rm {train}}}\times {n}_{{\rm {train}}}}$ the kernel matrix on drug pairs in training set

1: ${{\bf{K}}}_{x}^{{\rm {test}}}\in {{\mathbb{R}}}^{{n}_{{\rm {train}}}\times {n}_{{\rm {test}}}}$ the kernel matrix on drug pairs between training and test sets

2: *λ* regularisation parameter for KRR

3: ${[{S}_{i}]}_{i = 1}^{{n}_{{\rm {train}}}}$ list of fitted (BRAID) surface functions for training data

4: ${[{h}_{1}]}_{j = 1}^{{n}_{{\rm {test}}}}$ and ${[{h}_{2}]}_{j = 1}^{{n}_{{\rm {test}}}}$, lists of monotherapy (Hill) functions for the drugs in test data

5: ${[{C}_{1}]}_{j = 1}^{{n}_{{\rm {test}}}}$, ${[{C}_{2}]}_{j = 1}^{{n}_{{\rm {test}}}}$ lists of concentration arrays on which predictions should be made

6: $${\bf{M}}\leftarrow {({{\bf{K}}}_{x}^{{\rm {train}}}+\lambda {\bf{I}})}^{-1}$$ ⊳Training

7: $${\bf{Z}}\leftarrow {{\bf{MK}}}_{x}^{{\rm {test}}}$$

8: **if** use normalised output kernel **then**

9:  **S** ← sample training surfaces on normalised grid

10: **end if**

11: *T* ← [ ]    ⊳ *T* collects predictions at queried concentrations

12: **for** *t* ∈ [1, …, *n*_test_] **do**     ⊳ Loop for predicting

13:  *Q* ← candidate surfaces with *h*_1_[*t*] and *h*_2_[*t*] as monotherapies

14:  **if** use normalised output kernel **then**

15:   **Q** ← sample candidate surfaces *Q* on normalised grid defined on [0, 1] × [0, 1]

16:  **else if** use the original output kernel **then**

17:   **S** ← sample training set surfaces on test concentration grid based on *C*_1_[*t*] and *C*_2_[*t*]

18:   **Q** ← sample candidate surfaces *Q* on test concentration grid based on *C*_1_[*t*] and *C*_2_[*t*]

19:  **end if**

20:  $${{\bf{K}}}_{y}^{{\rm {test}}}={[\exp (-\gamma \parallel {{\bf{S}}}_{i}-{{\bf{Q}}}_{j}{\parallel }_{{\rm {F}}}^{2})]}_{i,j}$$; *i* ∈ [1, *n*_train_], *j* ∈ [1, *n*_candidates_]

21:  $i\leftarrow \mathop{{\mathrm{arg}}\,{\mathrm{max}}}\limits_{[1,{n}_{{\rm {candidates}}}]}{[{{\bf{K}}}_{y}^{{\rm {test}}}]}^{\top }{\bf{Z}}[:,t]$ ⊳ **Z**[:, *t*] denotes *t*th column of **Z**

22:  **if** use normalised output kernel **then**

23:   add *Q*_*i*_ sampled at concentration grid based on *C*_1_[*t*] and *C*_2_[*t*] to *T*

24:  **else if** use the original output kernel **then**

25:   add **Q**_*i*_ to *T*

26:  **end if**

27: **end for**

28: **return** *T*

The pseudocode of the approach can be found in Algorithm 1. Our approach assumes fitted monotherapy functions (Hill equations in our experiments) for the drugs in the test set, and also fitted surface functions for the training data (BRAID surfaces in our experiments); computing these can be seen as a preprocessing step. We note that for the new combo scenario, all these Hill equations are equations of the drugs in the training set, but in the new drug scenario, the Hill equations also contain equations for previously unseen drugs.

Training of our model is done based on the closed-form solution and consists of computing ${({{\bf{K}}}_{x}+\lambda {{\bf{I}}}_{n})}^{-1}{k}_{x}(X,x)$ from Eq. (2) (lines 6 and 7). For predicting, we consider candidate set optimisation. Here, we take advantage of the domain knowledge and the assumption that the monotherapy functions for the drugs in the test set are known. Now, the candidates can be created by directly using the parameters available in the Hill equations and by generating various combinations of the other required parameters; for the BRAID function the parameters *R*_3_ and *κ*. Finally, one of the candidate surfaces is chosen as the final prediction following Eq. (2) (lines 20 and 21). It is good to note that in order to obtain predictions for a drug outside of the training set, the model requires no re-training, but only the Hill equation of the drug in question.

In this work, for our experiments, we choose *k*_*x*_ to be defined as ${k}_{x}(({d}_{1},{d}_{2}),({d}_{1}^{{\prime} },{d}_{2}^{{\prime} }))={k}_{d}({d}_{1},{d}_{1}^{{\prime} }){k}_{d}({d}_{2},{d}_{2}^{{\prime} })$ with *k*_*d*_ acting on the MACCS fingerprints to be Tanimoto kernel^43^. As discussed, for the output surfaces, we consider the RBF kernel, which conforms to the assumption of having the output kernel being normalised. We use the parameter $\gamma =\frac{1}{2{\sigma }^{2}}$ with *σ* equal to the mean of distances between the surfaces in the training set. Results comparing different output kernels can be found from the supplementary material.

#### Remark 1

For simplicity, we have chosen to use the pairwise kernel defined as ${k}_{x}(({d}_{1},{d}_{2}),({d}_{1}^{{\prime} },{d}_{2}^{{\prime} }))={k}_{d}({d}_{1},{d}_{1}^{{\prime} }){k}_{d}({d}_{2},{d}_{2}^{{\prime} })$. It is not invariant to the order of the drugs, and thus, in practice results in the requirement of “doubling” the data in training by including both orders.

Formulations for invariant kernels exist (see e.g. ref. ^25^), but we observed negligible differences in observed performance between the kernels (Table 3).

**Table 3:** Minimum, average and maximum differences of Pearson correlations over all the cell lines on the O’Neil data results, when a non-invariant input kernel was used along with the data doubling, or an invariant kernel was considered

	New combo	New drug
Minimum difference	0.0	4.2e−05
Average difference	0.000668	0.001973
Maximum difference	0.004697	0.008049

#### Concentration normalisation to obtain a normalised surface kernel

**Fig. 7:**
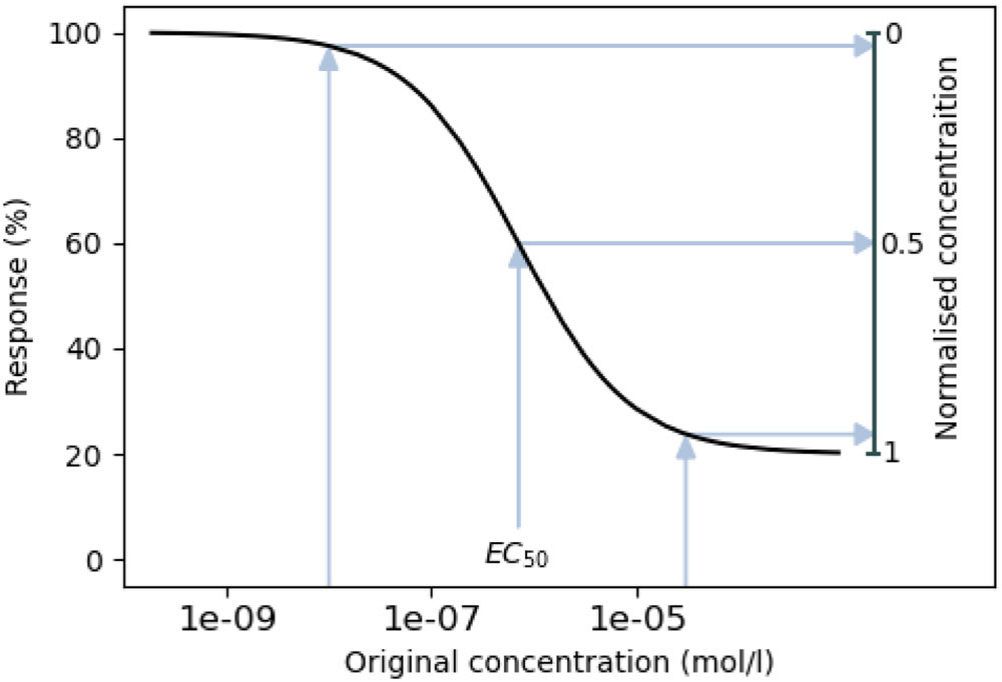
Illustration of the concentration normalisation procedure to [0, 1] range. The concentrations too low to elicit a response are mapped to zero, EC_50_ concentration is mapped to 0.5, and concentrations close to maximal effect are mapped close to 1.

The main idea of our proposed surface normalisation scheme is to standardise the concentration measurements across the different drugs so that all the concentrations are in the same range, and the surface comparisons can be made more easily. To this end, we map the dose concentrations with the help of Hill equations, individually for each drug in each cell line, to the range [0, 1], where 0 intuitively stands for “no effect”, and 1 stands for “maximal effect” (see Fig. 7). More concretely, the concentration normalisation transformation CN can be written as

$${\rm {CN}}(c| {R}_{0},{R}_{{\rm {max}}},\tau ,{\rm {E{C}}}_{50})=\frac{h(c| {R}_{0},{R}_{{\rm {max}}},\tau ,{\rm {E{C}}}_{50})-{R}_{0}}{{R}_{{\rm {max}}}-{R}_{0}}$$

in which *c* is the original concentration, and *h* is the Hill equation with parameters *R*_0_, *R*_max_, *τ* and EC_50_ (i.e. baseline response, maximal response, slope parameter and half maximal effective concentration). The normalisation formula uses the same *R*_0_ and *R*_max_ as the Hill equation.

After the concentration normalisation, the values [0, 1] can be seen to correspond to each other over all the different drugs—unlike in non-normalised cases where a concentration might yield a very different response from two different drugs. With all drug concentrations normalised, comparing different surfaces is easy in a common grid of values from [0, 1] × [0, 1] (even if for different drugs and cell lines they map to different original concentrations). Due to the nature of the normalisation scheme, it can be expected that this common drug grid in all surfaces will focus especially on areas where the potential synergy or antagonism can best be captured (as illustrated in the second part of Fig. 1).

### Experimental setup

We compare our comboKR (available at https://github.com/aalto-ics-kepaco/comboKR) to two baselines: LTR^26,44^ for predicting individual response values, and PIICM^25,45^ as another surface-valued prediction method based on Gaussian processes. With our comboKR, we consider both the original and normalised surfaces in output kernel computations: the former case is denoted by comboKR r.

LTR is a polynomial regression model for scalar-valued prediction that exploits higher order interactions between the views in predictions. As input for a prediction, it takes the two drugs and their concentrations. As comboKR considers kernels on input data, for LTR we use the empirical features^46^ from Tanimoto kernel evaluated on the MACCS fingerprints. As for the dose-concentrations, the LTR method represents them as one-hot encoded vectors.

PIICM is a surface-valued approach based on Gaussian process regression, that uses latent GP model from bayesynergy R package^47^ to model the drug response surfaces.^25^ conducted the experiments with the O’Neil dataset also considered in our work. Thus, with the O’Neil dataset we use their training-test split in the new combo scenario, with their original surface modelling and data normalisation scheme, both of which differ from ours. Contrary to^25^, to adhere within our setting, we train separate models for each cell line. With NCI-ALMANAC dataset we instead use the same BRAID surfaces as the baseline models as in our comboKR. Like in ref. ^25^, we consider a subset of concentrations to represent the surfaces since the method is computationally too heavy to run with a full set of concentrations in the data (over 60 unique dose values), and the BRAID surfaces are sampled at these concentrations as training data to the model. As the original PIICM concentration normalisation also resulted in a [0, 1] interval, when using the BRAID surface model, we also consider our novel normalisation scheme.

Yet, we observed that the memory requirements for the PIICM method to run with NCI-ALMANAC dataset in our setting were infeasible, as the number of drugs considered is relatively large. In order to run the method, we forced it to consider a simplified form of the drug combination covariance matrix—we call this PIICM^*^. While this might put the method at a disadvantage, in practice, we observe that in the new combo setting its performance is close to LTR, which is close to the results obtained in ref. ^25^. Moreover, we observed in small-scale experiments that our modification performs comparably to, or even slightly improves, the original parametrisation (see details in Supplementary Material). The PIICM^*^ outputs predictions on the chosen concentration grid. To obtain the final PIICM^*^ predictions, we interpolate with Nadaraya–Watson kernel regression from this set of concentrations to the concentrations at which the test surfaces are measured at.

The parameters for the models are chosen with cross-validation, taking into account the specifics of the predictive scenario in each split. For our comboKR, *λ* is chosen from 1e-2, 5e-2, 1e-1, 5e-1, while in LTR, the order is considered 3 or 5, and rank 10 or 20. PIICM^*^ and PIICM drug rank is cross-validated over 5, 25, 50, 75 and 100. In comboKR, the normalised grid is a 11 × 11 grid, based on normalised concentrations [0, 0.1, 0.2, …, 0.9, 0.999]. In all models, the training data is “doubled”, i.e. both orders of drug pairings are included separately in the training set.

#### Predictive scenarios

We consider predictions in two scenarios:

- *New drug*: one of the drugs in the combination queried at the test stage has not been seen in any combination during the training stage. Monotherapy responses of the novel drug are assumed to be available during the training stage.
- *New combo*: the drug combination queried at the test stage has not been seen in training; however, both single drugs may have been seen in the combinations encountered during training.

In the new combo setting, the data in each cell line is divided evenly into five folds, of which one is used as test fold, and the remaining folds are used in training and validation (with O’Neil data we use the same split as in ref. ^25^). In the new drug setting, the test fold contains all surfaces where either drug is one of ten (NCI-ALMANAC) or four (O’Neil) randomly chosen as the new drug. The four validation folds are obtained similarly among the rest of the data used in the training, always with randomly chosen drugs. Clearly, the new combo setting is the easiet of the two prediction scenarios. We note that in both scenarios, no drug-dose combination response values of a surface queried at the test point are available in training. The task is to predict all the combination responses, instead of filling missing values inside a matrix with known entries.

In the more challenging new drugs scenario, the drug features and monotherapy responses for the novel drug are assumed to be available already in the training stage. For most of the standard models, including the LTR baseline, it means that at any time a new drug is to be included in testing, the model would need to be re-trained with the additional information. This can be very costly. An advantage of our proposed comboKR method is that this kind of re-training is not needed, and the new monotherapy response data can be introduced to the model during the test stage when the candidate surfaces are being built. In this scenario, the relevant monotherapy responses of the new drugs in the test set are included to augment the LTR training set.

We note that the PIICM method^25^ is based on Gaussian processes, modelling the drug interaction surfaces and without considering any drug (or cell line) features when making predictions. Thus, it cannot generalise to data outside of the training set in the new drug scenario and cannot be applied there.

#### Obtaining the drug interaction surfaces

**Fig. 8:**
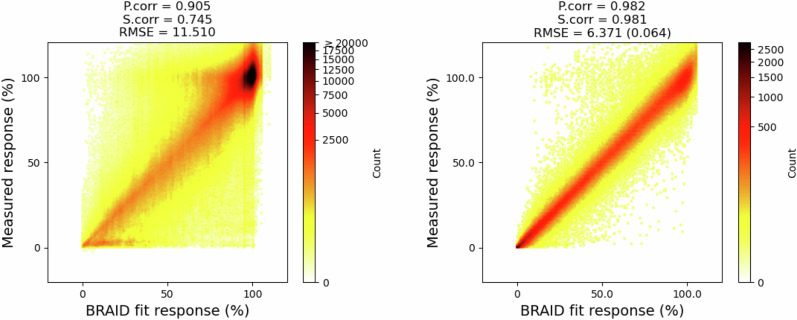
Density plots of the measured responses compared to the responses sampled from the fitted BRAID surface models at the same concentrations, over the full NCI-ALMANAC data (left) and full O’Neil data (right). As the viability response values in O’Neil are in range [0, 1], for better comparison to NCI-ALMANAC, they have been scaled to [0, 100] here. For RMSE in O’Neil, the first value is for scaled responses, the second value in parenthesis is for the original data.

As a preprocessing step for surface-based approaches (PIICM^*^ and our proposed comboKR), we fit the BRAID drug interaction surfaces to the available combo data with the synergy package^38^. Due to the scarcity of the monotherapy responses in the NCI-ALMANAC combination data, the relevant monotherapy responses available in NCI-60 were used to augment the combo data. Hill equations fitted to the monotherapy data are used in building the normalised output kernels. Overall, the average Pearson correlation of the fitted BRAID surfaces sampled at the measurement positions to the ground truth combination measurements was 0.9037 ± 0.0169 averaged over the cell lines, 0.905 over all of them in NCI-ALMANAC dataset, and 0.980 ± 0.015 averaged over the cell lines, and 0.982 over all of them in O’Neil dataset (see Fig. 8 for density plots).

**Fig. 9:**
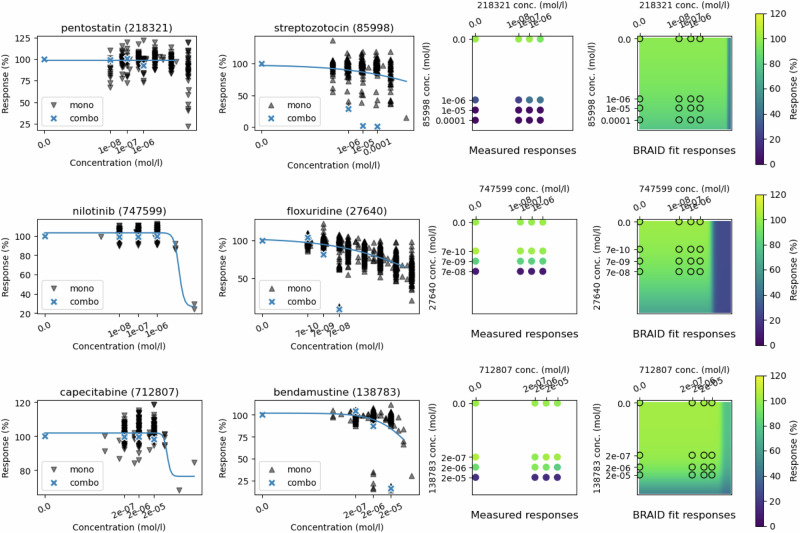
Examples of BRAID surfaces not fit well to the NCI-ALMANAC dataset. On top: cell line HL-60(TB), drugs pentostatin (id 218321) and streptozotocin (id 85998). Middle: cell line NCI-H226, drugs nilotinib (id 747599) and floxuridine (id 27640). Bottom: cell line OVCAR-8, drugs capecitabine (id 712807) and bendamustine (id 138783). The two first plots in each row show the NCI-60 monotherapy responses in black, with blue crosses highlighting the NCI-ALMANAC monotherapy response values, while the curve displays the responses from the Hill equation from the BRAID model. Third column displays the full combo data matrix, and the final column shows the fitted BRAID surface.

It can be expected that large amount of of the discrepancies are due to surface model filtering out noise from the measurements. However, some errors in fitting to the NCI-ALMANAC dataset are due to the conflicts between it and and NCI-60 dataset. Examples of this are provided in Fig. 9, where monotherapy data follows a clear trend and the monotherapy entries in combo data are outliers.

#### Candidate surfaces for comboKR

The simplest way to solve the pre-image problem (i.e. finding the best *y* for prediction *g*(*x*) in ${{\mathcal{H}}}_{{\mathcal{Y}}}$) for the comboKR problem (2), is to consider a candidate set where all elements are tested out, and the one giving the highest score is selected as the prediction. In both of the predictive scenarios, we assume that the monotherapies of the drugs are available. Thus, we can easily create relevant surface candidates with the surface model by only varying the parameters *κ* and *E*_max_ that are related to the behaviour of the drug combination. In experiments, the candidates are generated by sampling *κ* from [−1.5, −1, −0.5, −0.1, 0.01, 0.1, 0.5, 1, 2, 10] (to capture various surface interaction profiles), and *E*_max_ from around the maximum values of the individual drug responses.

#### Performance metrics

The test sets in both predictive settings consist of drug-dose interaction surfaces that have been sampled at various differing 4 × 4 grids. The predictions at those concentrations were obtained as follows: in LTR they are directly predicted, in PIICM they are interpolated from the predictions in the normalised grid, and in our comboKR they are sampled from the predicted BRAID surface. Two synergy scores (Bliss and Loewe) are calculated based on these ground truth and predicted 4 × 4 matrices.

We report Pearson correlations of the predictions (both for raw responses and summary synergy scores) calculated for each cell line separately. Within a cell line, both the predictions and ground truth labels are vectorised, and the correlation is calculated between the two vectors. The same procedure is used when investigating different tissue types and drug combination types: all the ground truth and predicted responses within the group in a cell line are vectorised to compute the Pearson correlation.

Furthermore, we compare all the methods to each other on the level of individual surfaces, by reporting how often in the test set each of the methods obtained the best prediction for a given surface with respect to the mean squared error—Pearson correlation not being applicable if the matrix of values that is sampled from the predicted surface is constant. This happens most often in modified PIICM, but also sometimes in both comboKR versions.

## Supplementary information

Supplementary Material

## Data Availability

The NCI-60 and NCI-ALMANAC datasets^7^ used are publicly available at https://wiki.nci.nih.gov/display/NCIDTPdata/NCI-60+Growth+Inhibition+Dataand https://wiki.nci.nih.gov/display/NCIDTPdata/NCI-ALMANAC. The O’Neil dataset is available as supplementary material associated with ref. ^40^.

## Code availability

The code for the comboKR method is available at https://github.com/aalto-ics-kepaco/comboKR. For the competing methods, we used codes made available for LTR at https://github.com/aalto-ics-kepaco/GO_LTR and adapted PIICM from https://github.com/ltronneb/PIICM/. The software for the BRAID surfaces is available from https://github.com/djwooten/synergy/.
