# Supplementary material for: Predicting drug combination response surfaces
Source: NPJ Drug Discov. 2025 Feb 3;2:2. doi: 10.1038/s44386-024-00004-z (PMC13267126; doi:10.1038/s44386-024-00004-z)
Supplement: Supplementary file 1 — Supplementary Material [file 44386_2024_4_MOESM1_ESM.pdf]

Predicting drug combination response surfaces Supplementary Material

Riikka Huusari[1], Tianduanyi Wang[1,2], Sandor Szedmak[1], Tero Aittokallio[2,3,4], and Juho Rousu[1]

[1]Department of Computer Science, Aalto University, Espoo, Finland
[2]Institute for Molecular Medicine Finland FIMM, HiLIFE, University of Helsinki, Helsinki, Finland
[3]Institute for Cancer Research, Department of Cancer Genetic, Oslo University Hospital, Norway
[4]Centre for Biostatistics and Epidemiology (OCBE), Faculty of Medicine, University of Oslo, Norway

The NCI-ALMANAC and O'Neil datasets

Supplementary Figure 1 shows the amounts of the 3×3 dose-response matrices for each cell line in the NCI-ALMANAC dataset. Supplementary Figure 2 displays the amounts of the monotherapy responses from NCI-60 dataset that were used. Additionally, Supplementary Figure 3 shows the amounts of the 4×4 dose-response matrices for each cell line in the O'Neil dataset.

Supplementary Figure 4 shows the full distribution of in how many surfaces concentration combinations are present. Majority of the dose combinations can be found in very few surfaces. The same is shown for O'Neil dataset in Supplementary Figure 5.

The tissue types in the NCI-ALMANAC dataset and lists of cell lines belonging to them are listed in Supplementary Table 1. Similarly, the drugs in the dataset and their types are listed in Supplementary Table 2.

The tissue types in the O'Neil dataset and lists of cell lines belonging to them are listed in Supplementary Table 3. Similarly, the drugs in the dataset and their types are listed in Supplementary Table 4.

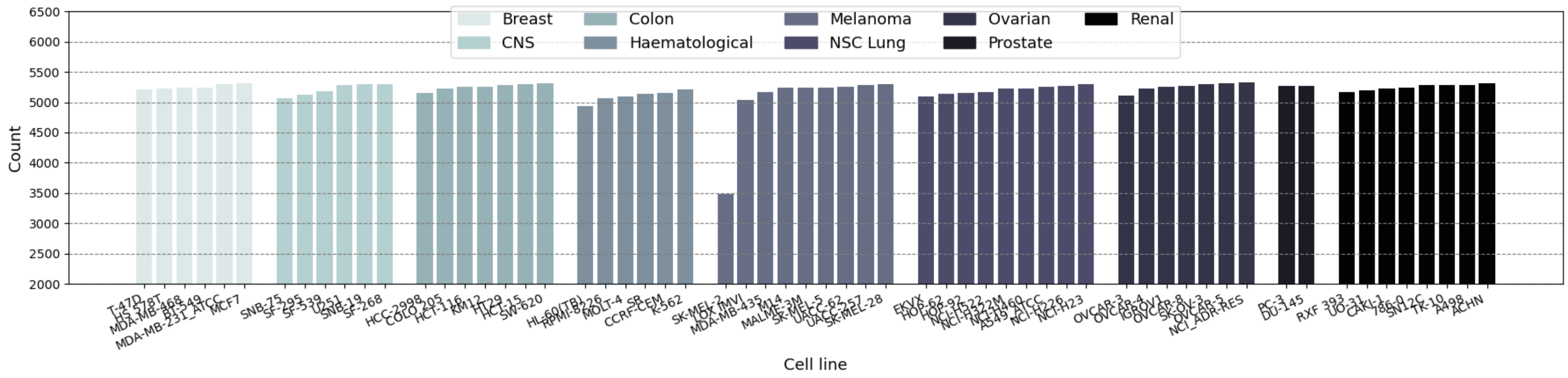

Supplementary Figure 1: Counts of the 3×3 dose-response measurement matrices (i.e. dose-response surfaces) in the processed NCI-ALMANAC dataset per cell line.

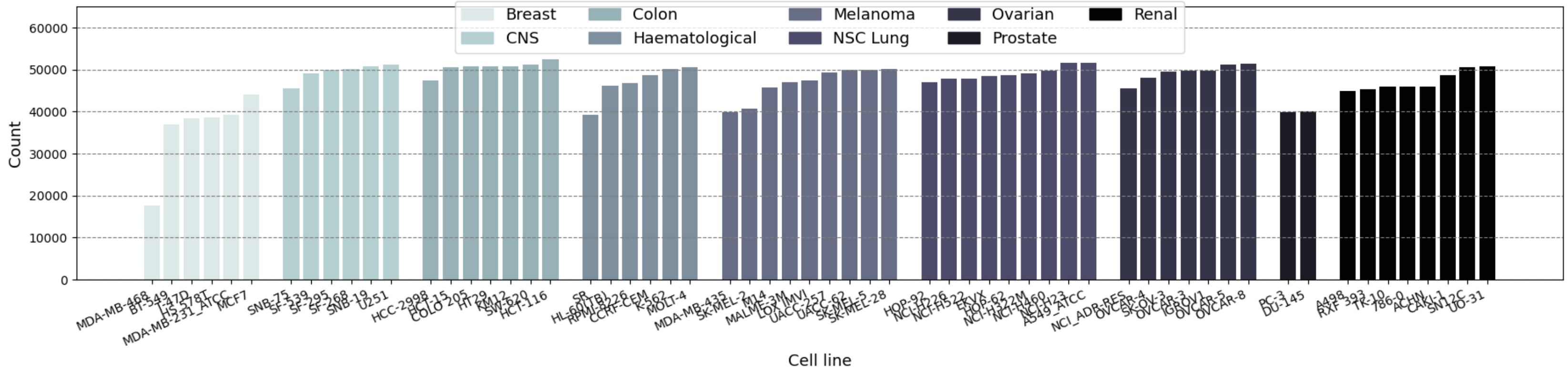

Supplementary Figure 2: Counts of individual dose-response measurements from the NCI-60 dataset used to assist in fitting the BRAID surfaces.

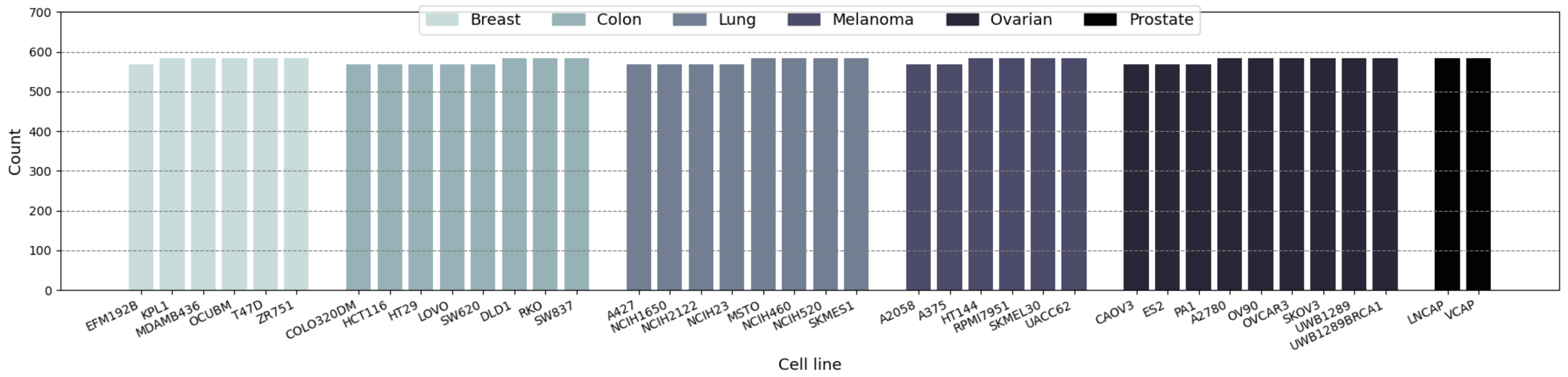

Supplementary Figure 3: Counts of the 4×4 dose-response measurement matrices (i.e. dose-response surfaces) in the processed O'Neil dataset per cell line.

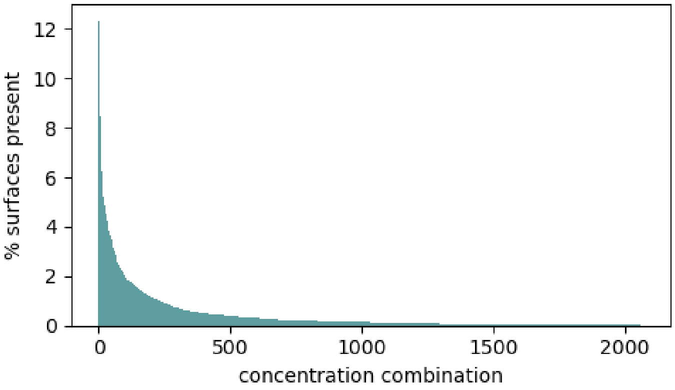

Supplementary Figure 4: Distribution on how many surfaces a concentration combination is present in (as percentage), in the NCI-ALMANAC drug combo dataset. The dose combinations are sorted in descending order w.r.t the amount of surfaces they are found in.

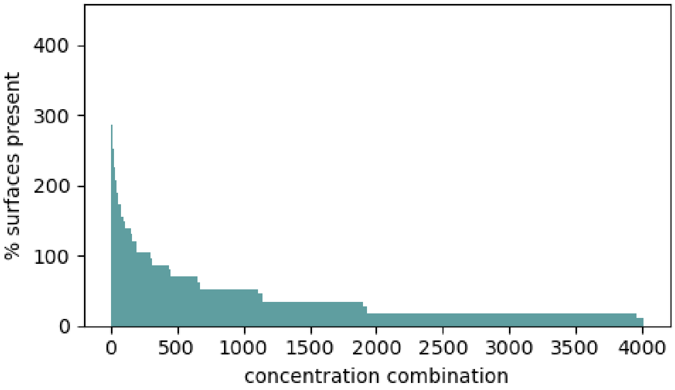

Supplementary Figure 5: Distribution on how many surfaces a concentration combination is present in (as percentage), in the O'Neil drug combo dataset. The dose combinations are sorted in descending order w.r.t the amount of surfaces they are found in.

Supplementary Table 1: The tissue types in NCI-ALMANAC dataset and the cell lines belonging to them.

Tissue type	Count	Cell line names
Breast	6	BT-549, HS 578T, MCF7, MDA-MB-231/ATCC, MDA-MB-468, T-47D
CNS	6	SF-268, SF-295, SF-539, SNB-19, SNB-75, U251
Colon	7	COLO 205, HCC-2998, HCT-116, HCT-15, HT29, KM12, SW-620
Haematological	6	CCRF-CEM, HL-60(TB), K-562, MOLT-4, RPMI-8226, SR
Melanoma	9	LOX IMVI, M14, MALME-3M, MDA-MB-435, SK-MEL-2, SK-MEL-28, SK-MEL-5, UACC-257, UACC-62
NSC Lung	9	A549/ATCC, EKVX, HOP-62, HOP-92, NCI-H226, NCI-H23, NCI-H322M, NCI-H460, NCI-H522
Ovarian	7	IGROV1, NCI/ADR-RES, OVCAR-3, OVCAR-4, OVCAR-5, OVCAR-8, SK-OV-3
Prostate	2	DU-145, PC-3
Renal	8	786-0, A498, ACHN, CAKI-1, RXF 393, SN12C, TK-10, UO-31

Supplementary Table 2: The drug types in NCI-ALMANAC dataset

Drug id	Drug name	Drug type	Drug id	Drug name	Drug type
740	methotrexate	Chemotherapy	246131	valrubicin	Chemotherapy
750	busulfan	Chemotherapy	256439	idarubicin	Not Used
752	thioguanine	Chemotherapy	256942	epirubicin	Not Used
755	mercaptopurine	Chemotherapy	266046	oxaliplatin	Chemotherapy
762	Mechlorethamine hydrochloride	Chemotherapy	279836	mitoxantrone	Chemotherapy
1390	allopurinol	Other	296961	amifostine	Chemotherapy
3053	dactinomycin	Chemotherapy	362856	temozolomide	Chemotherapy
3088	chlorambucil	Chemotherapy	369100	imiquimod	Other
6396	thiotepa	Chemotherapy	409962	carmustine	Chemotherapy
8806	melphalan	Chemotherapy	606869	clofarabine	Chemotherapy
9706	Triethylenemelamine	Chemotherapy	608210	vinorelbine	Chemotherapy
13875	altretamine	Chemotherapy	609699	topotecan	Chemotherapy
14229	mepacrine	Chemotherapy	613327	gemcitabine	Chemotherapy
18509	5-aminolevulinic-acid	Other	628503	docetaxel	Chemotherapy
19893	5-fluorouracil	Chemotherapy	673596	SN-38	Chemotherapy
24559	plicamycin	Chemotherapy	681239	bortezomib	Chemotherapy
25154	pipobroman	Chemotherapy	686673	nelarabine	Chemotherapy
26271	cyclophosphamide	Chemotherapy	698037	pemetrexed	Chemotherapy
26980	mitomycin-C	Chemotherapy	701852	vorinostat	Chemotherapy
27640	floxuridine	Chemotherapy	702294	estramustine-phosphate	Chemotherapy
32065	hydroxyurea	Chemotherapy	707389	eribulin	Not Used
34462	uracil-mustard	Chemotherapy	712807	capecitabine	Chemotherapy
38721	mitotane	Chemotherapy	713563	exemestane	Other
45388	dacarbazine	Chemotherapy	715055	gefitinib	Targeted therapy
45923	methoxsalen	Chemotherapy	718781	erlotinib	Targeted therapy
49842	vinblastine	Chemotherapy	719276	fulvestrant	Other
63878	cytarabine	Chemotherapy	719344	anastrozole	Chemotherapy
66847	thalidomide	Other	719345	letrozole	Other
67574	vincristine	Chemotherapy	719627	celecoxib	Chemotherapy
71423	Megestrol acetate	Other	721517	zoledronic-acid	Other
77213	procarbazine	Chemotherapy	732517	dasatinib	Targeted therapy
79037	lomustine	Chemotherapy	733504	everolimus	Targeted therapy
82151	daunorubicin	Chemotherapy	737754	pazopanib	Targeted therapy
85998	streptozotocin	Chemotherapy	743414	imatinib	Targeted therapy
92859	arsenic-trioxide	Chemotherapy	745750	lapatinib	Targeted therapy
102816	azacitidine	Chemotherapy	747599	nilotinib	Targeted therapy
105014	cladribine	Chemotherapy	747971	sorafenib	Targeted therapy
109724	ifosfamide	Chemotherapy	747972	lenalidomide	Other
118218	fludarabine	Chemotherapy	747973	ixabepilone	Other
119875	cisplatin	Chemotherapy	747974	raloxifene	Other
122758	tretinoin	Other	749226	abiraterone	Chemotherapy
122819	teniposide	Chemotherapy	750690	sunitinib	Targeted therapy
123127	doxorubicin	Chemotherapy	753082	vemurafenib	Targeted therapy
125066	bleomycin	Chemotherapy	754143	romidepsin	Other
125973	paclitaxel	Chemotherapy	754230	pralatrexate	Chemotherapy
127716	decitabine	Chemotherapy	755986	vismodegib	Targeted therapy
138783	bendamustine	Chemotherapy	756645	crizotinib	Targeted therapy
141540	etoposide	Chemotherapy	757441	axitinib	Targeted therapy
169780	dexrazoxane	Other	760766	vandetanib	Targeted therapy
180973	tamoxifen	Other	761431	vemurafenib	Targeted therapy
218321	pentostatin	Chemotherapy	761432	cabazitaxel	Chemotherapy
226080	sirolimus	Other	763371	ruxolitinib	Targeted therapy
241240	carboplatin	Chemotherapy			

Supplementary Table 3: The tissue types and the cell lines in O'Neil dataset belonging to them.

Tissue type	Count	Cell line names
Breast	6	EFM192B, KPL1, MDAMB436, OCUBM, T47D, ZR751
Colon	8	COLO320DM, DLD1, HCT116, HT29, LOVO, RKO, SW620, SW837
Lung	8	A427, MSTO, NCIH1650, NCIH2122, NCIH23, NCIH460, NCIH520, SKMES1
Melanoma	6	A2058, A375, HT144, RPMI7951, SKMEL30, UACC62
Ovarian	9	A2780, CAOV3, ES2, OV90, OVCAR3, PA1, SKOV3, UWB1289, UWB1289BRCA1
Prostate	2	LNCAP, VCAP

Supplementary Table 4: The drug types in O'Neil data

Name in dataset	Drug name	Drug type	Name in dataset	Drug name	Drug type
5-FU	5-fluorouracil	Chemotherapy	MK-8669	Ridaforolimus	Targeted therapy
ABT-888	Veliparib	Targeted therapy	MK-8776	MK-8776	Targeted therapy
AZD1775	Adavosertib	Targeted therapy	MRK-003	MRK-003	Targeted therapy
BEZ-235	Dactolisib	Targeted therapy	Metformin	Metformin	Other
Bortezomib	Bortezomib	Chemotherapy	Methotrexate	Methotrexate	Chemotherapy
Carboplatin	Carboplatin	Chemotherapy	Mitomycine	Mitomycine	Chemotherapy
Cyclophosphamide	Cyclophosphamide	Chemotherapy	Oxaliplatin	Oxaliplatin	Chemotherapy
Dasatinib	Dasatinib	Targeted therapy	PD325901	Mirdametinib	Targeted therapy
Dexamethasone	Dexamethasone	Chemotherapy	Paclitaxel	Paclitaxel	Chemotherapy
Dinaciclib	Dinaciclib	Targeted therapy	SN-38	SN-38	Chemotherapy
Doxorubicin	Doxorubicin	Chemotherapy	Sorafenib	Sorafenib	Targeted therapy
Erlotinib	Erlotinib	Targeted therapy	Sunitinib	Sunitinib	Targeted therapy
Etoposide	Etoposide	Chemotherapy	Temozolomide	Temozolomide	Chemotherapy
Gemcitabine	Gemcitabine	Chemotherapy	Topotecan	Topotecan	Chemotherapy
L778123	L778123	Targeted therapy	Vinblastine	Vinblastine	Chemotherapy
Lapatinib	Lapatinib	Targeted therapy	Vinorelbine	Vinorelbine	Chemotherapy
MK-2206	MK-2206	Targeted therapy	Zolinza	Vorinostat	Chemotherapy
MK-4541	MK-4541	Targeted therapy	geldanamycin	Geldanamycin	Chemotherapy
MK-4827	Niraparib	Targeted therapy			

PIICM modification

In order to assess the suitability of our PIICM modification (PIICM*), we performed a small scale experimental comparison to the original parametrisation. To this end, we subsampled the data in cell line ”786-0” (chosen as it was first in alphabetical order), and chose either every third or every fourth drug from alphabetical order to be included – choosing every second already resulted to memory errors with original PIICM. The data was divided into training, validation and testing according to the new combo scenario. The partition sizes are shown in Supplementary Table 5.

Supplementary Table 5: Data sizes in PIICM modification test runs.

Setting	#drugs	#surfaces	n_{tr}	n_{val}	n_{tst}
Every 3rd	35	569	341	113	115
Every 4th	26	296	177	59	60

PIICM takes as input a matrix **Y** collecting the response data, where a row corresponds to concentrations of the two drugs, and column to drug combination. While one can use as the columns just the drug combinations in training and test sets, in our modification we enumerate all combinations, to make the kronecker product structure of the covariance matrix make sense. Thus, the **Y** is larger in our modification. We compare to original PIICM parametrisation with both **Y** structures, and denote with PIICM† the version using **Y** as in our modification.

The different ranks used in PIICM cross-validation were selected from [10, 20, 25, 50, 75, 100, 150, 200, 250, 300, 400, 500]:

- In our modification, the rank is at most equal to number of drugs; thus, here the ranks were [10, 20, 25]
- In original parametrisation the drug rank is at most number of surfaces in the data. Thus, the ranks 300, 400 and 500 are only applicable to the setting where every third drug was selected.

For comparison, comboKR was cross-validated with λ values in [1e-5, 1e-4, 1e-3, 1e-2, 1e-1].

The results obtained on the test set are displayed in Supplementary Figure 6. It is clear that our modification does not negatively impact the PIICM results - in fact, it seems to offer a clear improvement compared to the original parametrisation. Running times of the final training-test cycle are displayed in Supplementary Table 6.

Supplementary Table 6: Running time of final training-test cycle

Method	running time (h:min:s)	
	every 3rd	every 4th
comboKR (both)	0:00:09.236669	0:00:02.603092
PIICM*	0:00:38.839474	0:00:24.557288
PIICM†	*memory error*	1:07:09.720523
PIICM	0:27:10.222010	0:15:39.983792

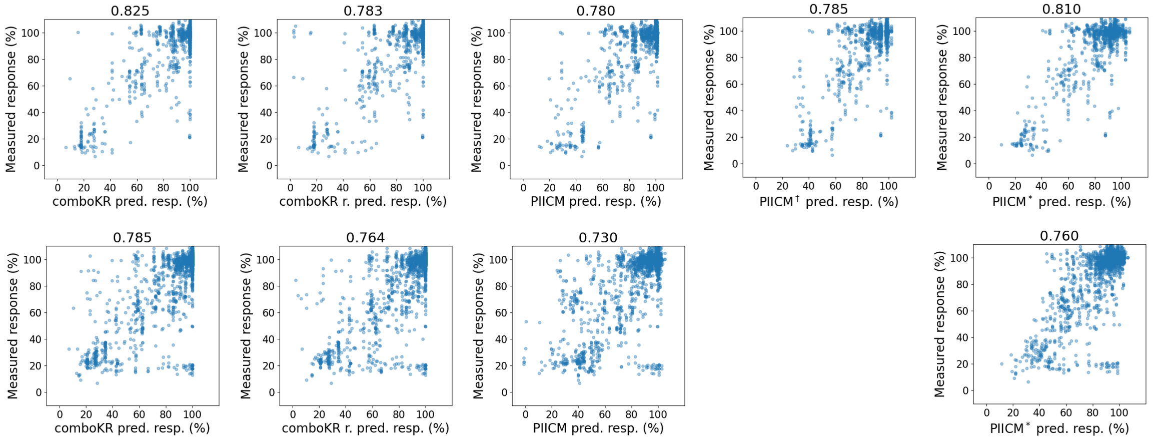

Supplementary Figure 6: Results of the small-scale experiments of PIICM modification comparison. The methods shown in the columns are comboKR, comboKR with normalised kernel, PIICM with original parametrisation, PIICM† with our modified input, and PIICM* with our modified input and parametrisation. Titles indicate the Pearson correlation to the measured responses. Top row: experimental setting "every fourth", bottom row: "every third". Memory error occurred for PIICM† with original parametrisation in the larger experiment.

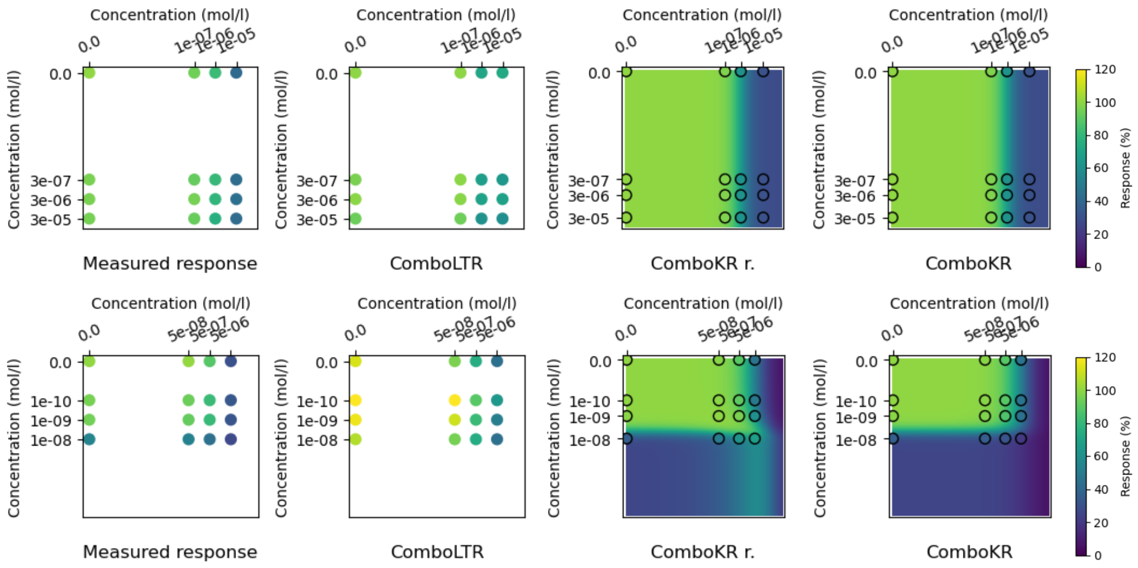

Supplementary Figure 7: Measured responses and example predictions from LTR and the two comboKR versions in the new drug setting from NCI-ALMANAC experiments. Top row shows combination of drugs thioguanine (id 752, horizontal axis) and lenalidomide (id 747972, on vertical axis), and bottom row of drugs triethylenemelamine (id 9706, on horizontal axis) and SN-38 (id 673596, on vertical axis), both of cell line 786-0.

Supplementary results

We illustrate the difference between the discrete LTR and continuous comboKR predictions in Supplementary Figure 7.

Pearson correlation tables

Tables 7 and 8 display mean and standard deviation of Pearson correlations, for both predicted response values, and the Bliss and Loewe synergies calculated from them. As ground truth, both the original measurements and the BRAID surfaces fit to the data and sampled at corresponding concentrations are considered. As ComboKR and PIICM* rely on the fitted surfaces instead of the raw measurements, it is natural that the performance with respect to BRAID is higher.

p-values for the results

Fig. 2 in the main manuscript presented selected p-values of the pairwise comparison of the results. Here, we present all the p-values from two-sample Kolmogorov–Smirnov test in Tables 9 and 10. In the new drug predictive scenario, differences between comboKR and other methods are always statistically significant.

New drug results as function of similarity to training set

Supplementary Figure 8 dispalays the mean squared error of the cobmoKR results for each individual predicted surface in the new drug setting, plotted as a function of maximal similarity to a training set drug pair.

Supplementary Table 7: Pearson correlations between the ground truth and predicted responses, as well as between Bliss and Loewe synergy scores of the ground truth and predicted responses, on the NCI-ALMANAC dataset. The results are averaged over all 60 cell lines (± standard deviation). The performance at test stage is measured to both original ground truth measurements, as well as to BRAID surfaces sampled at those concentrations.

(a) New combo scenario

	Responses	
	Measurements	BRAID
comboKR r.	0.848 ± 0.022	0.949 ± 0.014
comboKR n.	0.847 ± 0.024	0.948 ± 0.017
LTR	0.866 ± 0.014	0.870 ± 0.013
PIICM*	0.821 ± 0.037	0.914 ± 0.038

	Bliss synergy scores	
	Measurements	BRAID
comboKR	0.887 ± 0.021	0.953 ± 0.014
comboKR n.	0.886 ± 0.023	0.952 ± 0.017
LTR	0.911 ± 0.016	0.916 ± 0.013
PIICM*	0.881 ± 0.028	0.939 ± 0.026

	Loewe synergy scores	
	Measurements	BRAID
comboKR r.	0.865 ± 0.020	0.941 ± 0.016
comboKR n.	0.864 ± 0.023	0.940 ± 0.020
LTR	0.895 ± 0.016	0.898 ± 0.013
PIICM*	0.851 ± 0.036	0.917 ± 0.039

(b) New drug scenario.

	Responses	
	Groundtruth	BRAID
comboKR r.	0.792 ± 0.039	0.895 ± 0.029
comboKR n.	0.838 ± 0.044	0.936 ± 0.028
LTR	0.674 ± 0.033	0.692 ± 0.036

	Bliss synergy scores	
	Groundtruth	BRAID
comboKR	0.838 ± 0.035	0.906 ± 0.029
comboKR n.	0.881 ± 0.035	0.944 ± 0.025
LTR	0.836 ± 0.045	0.851 ± 0.038

	Loewe synergy scores	
	Groundtruth	BRAID
comboKR	0.801 ± 0.038	0.882 ± 0.034
comboKR n.	0.852 ± 0.042	0.927 ± 0.032
LTR	0.787 ± 0.050	0.799 ± 0.044

Supplementary Table 8: Pearson correlations between the ground truth and predicted responses, as well as between Bliss and Loewe synergy scores of the ground truth and predicted responses on the O'Neil dataset. The results are averaged over all 39 cell lines (± standard deviation). The performance at test stage is measured to both original ground truth measurements, as well as to BRAID surfaces sampled at those concentrations.

(a) New combo scenario

	Responses	
	Measurements	BRAID
ComboKR	0.938 ± 0.033	0.956 ± 0.020
ComboKR r.	0.923 ± 0.034	0.939 ± 0.023
LTR	0.925 ± 0.030	0.937 ± 0.020
PIICM	0.964 ± 0.023	0.973 ± 0.015

	Bliss synergy scores	
	Measurements	BRAID
ComboKR	0.918 ± 0.044	0.939 ± 0.030
ComboKR r.	0.899 ± 0.045	0.916 ± 0.034
LTR	0.903 ± 0.039	0.916 ± 0.029
PIICM	0.955 ± 0.030	0.963 ± 0.022

	Loewe synergy scores	
	Measurements	BRAID
ComboKR	0.854 ± 0.101	0.885 ± 0.086
ComboKR r.	0.823 ± 0.110	0.847 ± 0.101
LTR	0.832 ± 0.083	0.850 ± 0.073
PIICM	0.921 ± 0.059	0.933 ± 0.050

(b) New drug scenario

	Responses	
	Measurements	BRAID
ComboKR	0.924 ± 0.032	0.939 ± 0.026
ComboKR r.	0.782 ± 0.075	0.798 ± 0.074
LTR	0.797 ± 0.066	0.808 ± 0.063

	Bliss synergy scores	
	Measurements	BRAID
ComboKR	0.899 ± 0.043	0.915 ± 0.037
ComboKR r.	0.717 ± 0.090	0.735 ± 0.089
LTR	0.729 ± 0.089	0.740 ± 0.088

	Loewe synergy scores	
	Measurements	BRAID
ComboKR	0.836 ± 0.099	0.857 ± 0.092
ComboKR r.	0.580 ± 0.147	0.599 ± 0.146
LTR	0.615 ± 0.129	0.625 ± 0.128

Supplementary Table 9: Results of the two-sample Kolmogorov–Smirnov test on the different methods on NCI-ALMANAC dataset, on the response predictions as well as the synergy scores calculated on them for the two predictive scenarios. p-values less than 0.01 are highlighted with light grey, and those less than 0.05 with dark grey.

(a) Responses, new combo

	ComboKR	ComboKR r.	LTR
ComboKR r.	9.87e-01		2.55e-04
LTR	5.57e-04	2.55e-04	
PIICM*	5.57e-04	2.55e-04	6.10e-13

(b) Responses, new drug

	ComboKR	ComboKR r.	LTR
ComboKR r.	1.10e-08		1.70e-28
LTR	1.48e-31	1.70e-28	

(c) Bliss, new combo

	ComboKR	ComboKR r.	LTR
ComboKR r.	8.13e-01		2.25e-10
LTR	2.25e-10	2.25e-10	
PIICM*	5.13e-01	6.65e-01	1.31e-11

(d) Bliss, new drug

	ComboKR	ComboKR r.	LTR
ComboKR r.	2.25e-10		5.13e-01
LTR	3.65e-08	5.13e-01	

(e) Loewe, new combo

	ComboKR	ComboKR r.	LTR
ComboKR r.	8.13e-01		2.25e-14
LTR	6.10e-13	2.25e-14	
PIICM*	1.82e-01	2.76e-02	3.93e-15

(f) Loewe, new drug

	ComboKR	ComboKR r.	LTR
ComboKR r.	5.57e-11		2.67e-01
LTR	2.25e-10	2.67e-01	

Ablation study: choice of the output kernel

We investigated how choosing a different output kernel affects the comboKR results, and display them in Supplementary Figure 9.

Supplementary Table 10: Results of the two-sample Kolmogorov–Smirnov test on the different methods on the O'Neil dataset, on the response predictions as well as the synergy scores calculated on them for the two predictive scenarios. p-values less than 0.01 are highlighted with light grey, and those less than 0.05 with dark grey.

(a) Responses, new combo

	ComboKR	ComboKR r.	LTR
ComboKR r.	1.04e-03		9.11e-01
LTR	1.04e-03	9.11e-01	
PIICM*	3.19e-09	1.94e-13	1.72e-12

(b) Responses, new drug

	ComboKR	ComboKR r.	LTR
ComboKR r.	1.55e-15		2.52e-01
LTR	1.05e-16	2.52e-01	

(c) Bliss, new combo

	ComboKR	ComboKR r.	LTR	PIICM
ComboKR r.	1.25e-02		7.52e-01	
LTR	5.81e-03	7.52e-01		
PIICM	5.72e-10	1.94e-13	1.34e-11	

(d) Bliss, new drug

	ComboKR	ComboKR r.	LTR
ComboKR r.	1.55e-15		7.52e-01
LTR	1.55e-15	7.52e-01	

(e) Loewe, new combo

	ComboKR	ComboKR r.	LTR	PIICM
ComboKR r.	2.56e-02		9.11e-01	
LTR	1.04e-03	9.11e-01		
PIICM	3.21e-07	1.34e-11	5.72e-10	

(f) Loewe, new drug

	ComboKR	ComboKR r.	LTR
ComboKR r.	1.94e-13		5.62e-01
LTR	1.94e-13	5.62e-01	

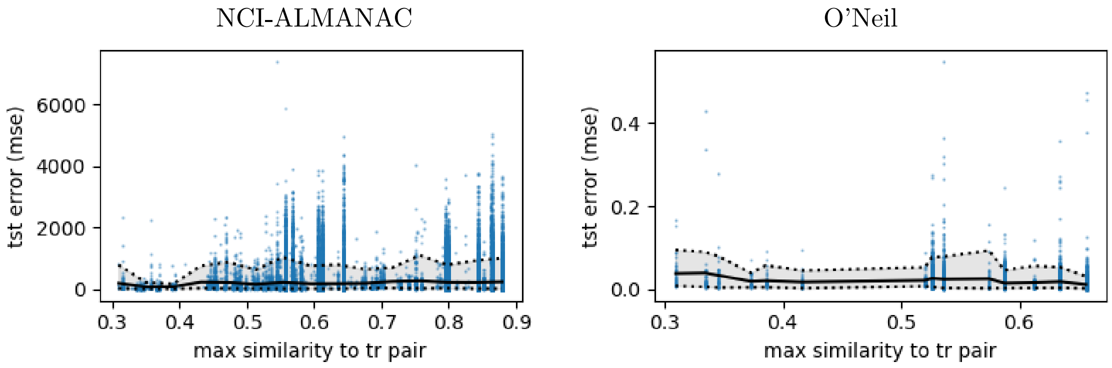

Supplementary Figure 8: The mean squared errors of the individual surface predictions in the NCI-ALMANAC (left) and O'Neil datasets (right), as functions of the maximal similarity to a training set drug pair. The solid line marks the average and dashed lines with filled-in background between them mark the 5 and 95 percentiles; for NCI-ALMANAC these calculations have been done after binning the data into 15 bins. Note that ALMANAC responses are mostly in [0, 100], while O'Neil responses range mostly in [0, 1].

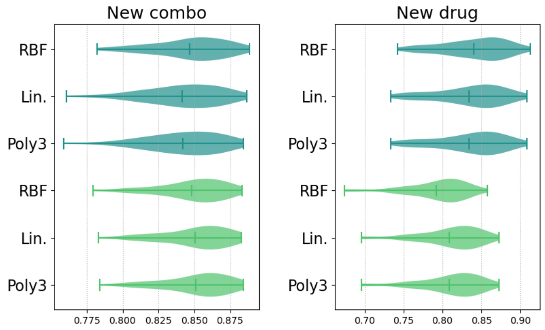

(a) NCI-ALMANAC dataset.

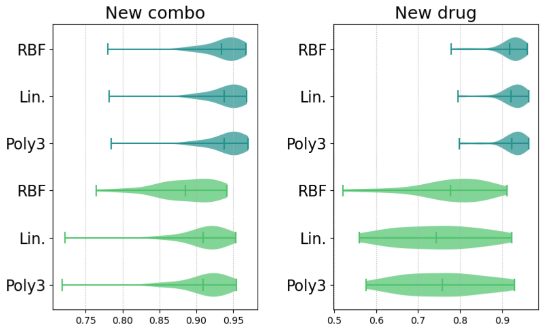

(b) O'Neil dataset.

Supplementary Figure 9: The Pearson correlation results of the comboKR (top three violins, darker colour) and comboKR r approaches (bottom three violins, lighter colour), in the new combo and new drug scenarios, with the three output kernels: RBF, linear, and polynomial kernel of degree 3.
